# Homologous recombination occurs frequently at innate GT microsatellites in normal somatic and germ cells in vivo

**DOI:** 10.1186/s12864-018-4758-y

**Published:** 2018-05-11

**Authors:** Jianbo Zheng, Heng Li, Qi Zhang, Lei Sun, Xiangfang Liu, Chen Luo

**Affiliations:** College of Life Sciences, Zhejiang University, Hangzhou, 310058 Zhejiang China

**Keywords:** Homologous recombination, Genome replication, GT microsatellites, Somatic cells, Germ cells

## Abstract

**Background:**

In somatic cells, homologous recombination (HR) is a rare event caused by eventual DNA double-strand breaks (DSBs). In contrast, germ cells show high frequency of HR caused by programmed DSBs. Microsatellites are prone to DSBs during genome replication and, thereby, capable of promoting HR. It remains unclear whether HR occurs frequently at microsatellites both in normal somatic cells and germ cells in a similar manner.

**Results:**

By examining the linkage pattern of multiple paternal and maternal markers flanking innate GT microsatellites, we measured HR at the GT microsatellites in various somatic cells and germ cells in a goldfish intraspecific heterozygote. During embryogenesis, the HR products accumulate gradually with the increase of the number of cell divisions. The frequency of HR at the GT microsatellites in advanced embryos, adult tissues and germ cells is surprisingly high. The type of exchanges between the homologous chromosomes is similar in normal advanced embryos and germ cells. Furthermore, a long GT microsatellite is more active than a short one in promoting HR in both somatic and germ cells.

**Conclusions:**

HR occurs frequently at innate GT microsatellites in normal somatic cells and germ cells in a similar manner.

**Electronic supplementary material:**

The online version of this article (10.1186/s12864-018-4758-y) contains supplementary material, which is available to authorized users.

## Background

Homologous recombination (HR) is a type of genetic recombination in which DNA sequences are exchanged between two similar or identical DNA molecules. HR in germ cells and in somatic cells is generally considered to be markedly different in several aspects, including function, initiating lesions and outcome [1]. HR in somatic cells is thought to be a rare event caused by eventual DNA double-strand breaks (DSBs), which frequently leads to non-crossover recombination and has potential detrimental effects due to loss of heterozygosity (LOH) [1–3]. In contrast, HR in germ cells is considered as a normal meiotic event initiated by programmed DSBs. This may result in crossover (CO) between homologous chromosomes and is essential for their faithful segregation at the meiosis I spindle to produce haploid gametes and increasing genetic diversity in progeny [1, 4]. Tandem-repeat DNA motifs of 1-6 nucleotides, named microsatellites or simple sequence repeats, are prone to DSBs due to replication stalling and slippage during genome replication [5–11], and, thereby, capable of promoting HR in the process of DNA synthesis. Since microsatellites are widely prevalent in eukaryotic genomes, it is relevant to know whether HR occurs frequently at microsatellites in both somatic and germ cells in a genome replication-dependent manner.

Repeat dinucleotides (GT)_n_ is one of the most common microsatellites and ubiquitously present in eukaryotic genomes [12–14]. Studies in cell cultures have shown that GT repeats can stimulate extrachromosomal recombination of transfected DNA [15–18]. An insertion of (GT)_n_ repeat in a yeast chromosome can significantly enhance reciprocal meiotic recombination [19, 20] and stimulate the formation of multiple crossovers [20]. Biochemical in vitro experiments have observed that recombinase proteins, such as bacteria RecA, yeast Rad51 and human hsRad51, preferentially bind to the GT repeat sequence [21, 22]. Bioinformatic analysis of recombination patterns across human chromosome 22 and their correlation with GT repeats density suggests that innate (GT)_n_ motif is associated with HR [23]. Therefore, we selected innate (GT)_n_ motifs for the in vivo study of whether HR at microsatellites has biological importance in both somatic and germ cells of vertebrates. Our results show that HR occurs frequently at innate (GT)_n_ motifs in a similar manner in somatic cells and germ cells.

## Results

### A goldfish intraspecific heterozygote provides an ideal animal system for detecting HR at GT repeats

**Fig. 1:**
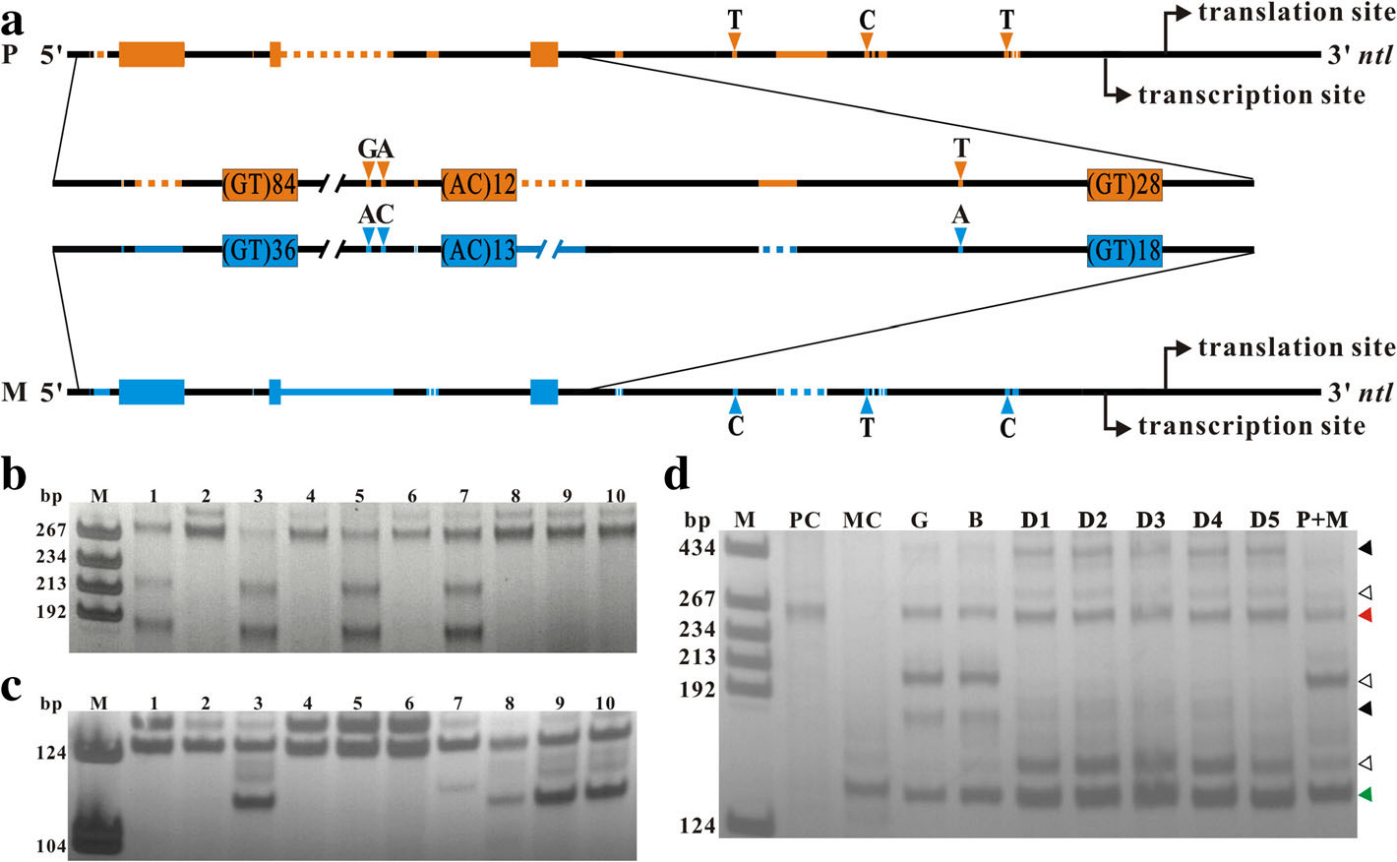
Position and instability of (GT)_n_ motifs in CGF and JGF goldfish strains. **a** Comparison of paternal and maternal promoters of *ntl* gene in goldfish intraspecific heterozyote. Black lines represent the conserved regions. Orange and blue boxes represent paternal and maternal (GT)_n_ motifs, respectively. Orange and blue lines represent the paternal and maternal specific inserted sequences, respectively. Orange and blue dotted lines represent the paternal and maternal specific deleted sequences, respectively. Orange and blue arrow heads indicate the paternal and maternal specific SNP sites, respectively. **b**, **c**, Gel electrophoretogram of PCR products amplified from CGF and JGF genome DNA, respectively. M, markers. 1-10, the number of the samples. **d** Gel electrophoretogram of PCR products of (GT)_n_ motif 1 in heterozygous embryos at different developmental stages. M, marker. PC, paternal control. MC, maternal control. G, gastrula stage embryos. B, bud stage embryos. D1-D5, 1-day to 5-day old embryos. P + M, mixed sample of equal paternal and maternal genomic DNA. Red and green arrow heads indicate the PCR products identical to paternal CGF and maternal JGF (GT)_n_ motif 1, respectively. Black and white arrow heads indicate the PCR products of (GT)_n_ motif 1 detected only in the embryo samples and detected in both the embryo and mix control samples, respectively

In a previous study, we identified an evolutionary conserved imperfect (GT)_n_ motif at the promoter of *no tail* (*ntl*) gene in different fish species [24]. Since there are many lines and subspecies with different genetic backgrounds, goldfish *ntl* promoter region containing the (GT)_n_ motif was employed for our study. To obtain different goldfish strains that contain distinguishable genetic markers, the flanking sequences of the (GT)_n_ motif from the genome of different goldfish strains were sequenced and compared. Fortunately, a Chinese goldfish (CGF) strain, *Carassius auratus auratus* and a Japanese goldfish (JGF) strain, *Carassius auratus cuvieri*, have diversified genetic markers in the flanking sequences of the (GT)_n_ motif. There is a 39 bp long indel in the upstream flanking sequence and two single nucleotide polymorphism (SNP) sites associated with a 3 bp long indel in the downstream flanking sequence (Fig. 1a). Moreover, there are two downstream short (GT)_n_ motifs which are also flanked by diversified sequences in the two goldfish strains (Fig. 1a). The three (GT)_n_ motifs in the upper, middle and downstream were named as (GT)_n_ motif 1, 2 and 3, respectively. The sequence direction of (GT)_n_ motif 2 is opposite to that of (GT)_n_ motif 1 and 3 in both strains. (GT)_n_ motif 1 is the longest of the three (GT)_n_ motifs in both CGF and JGF strains (Fig. 1a). Length polymorphisms of (GT)_n_ motif 1 were detected by PCR analyses in both CGF (Fig. 1b) and JGF strains (Fig. 1c). However, (GT)_n_ motif 1 is much longer in CGF strain than in JGF strain (Fig. 1b, c).

To examine HR at GT repeats in both somatic and germ cells, we generated an intraspecific heterozygote of goldfish by crossing a homozygous CGF male individual containing an imperfect (GT)_n_ motif 1 of about 168 bases with a homozygous JGF female individual containing an imperfect (GT)_n_ motif 1 of about 72 bases (Fig. 1 and Additional file 1: Figure S1, S2, S3). In this heterozygote, the parental-derived sequences and different HR sequences could be identified unequivocally by multiple paternal and maternal genetic markers flanking the (GT)_n_ motifs. The heterozygotes developed smoothly and mature heterozygous female and male mating reproduced normal offspring. Therefore, this goldfish intraspecific heterozygote provided an ideal animal system for detecting HR at GT repeats.

### HR occurs frequently at innate (GT)_n_ motif during mitosis in normal somatic cells in vivo

It is well known that HR is initiated by DSBs [1, 4], and DSBs formation in or near tandem repeats can elicit contraction and expansion of the tandem repeats during DSBs repair [7, 10, 11, 25]. We first examined whether DSBs occur at (GT)_n_ motif 1 during embryogenesis by analyzing the length of (GT)_n_ motif 1 at different developmental stages. PCR amplification with a pair of primers encompassing just the (GT)_n_ motif 1 detected various contracted and expanded products in all the genome samples from heterozygous embryos and in the equal mixed parent genome samples (Fig. 1d). Though similar PCR products were amplified from the heterozygous and the mixed parental samples (probably generated by template-switching in PCR reactions), some specific contracted and expanded PCR products were obtained only from the genome of heterozygote embryos (Fig. 1d), suggesting that DSBs were formed in or near (GT)_n_ motif 1 during embryogenesis in the heterozygotes.

**Fig. 2:**
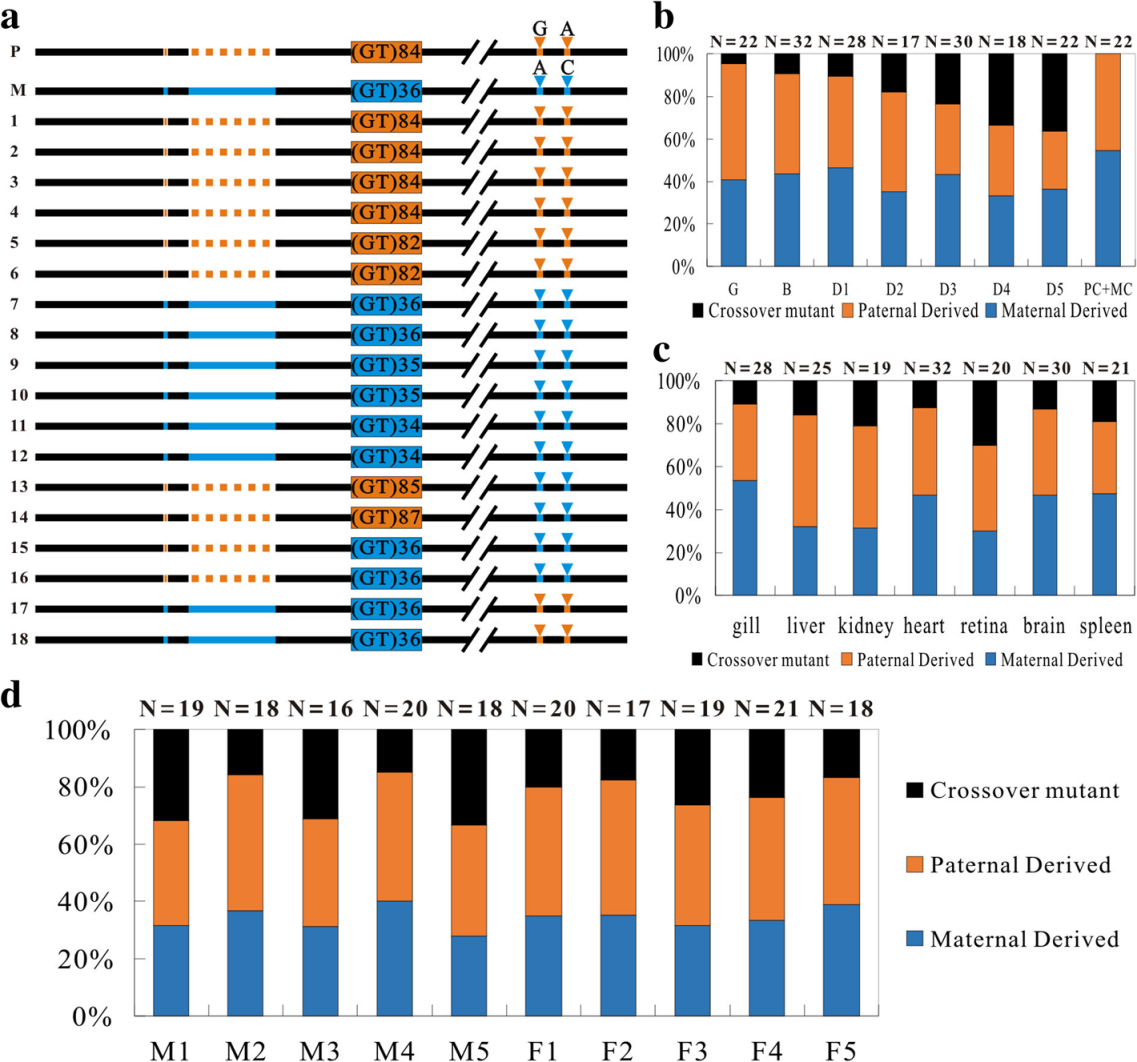
Mitotic homologous recombination at the (GT)_n_ motif1 region. **a** The linkage patterns of the paternal and maternal genetic makers flanking (GT)_n_ motif1 in 4-day old heterozygous embryos. Black lines represent the conserved regions. Orange lines, dotted lines and arrow heads represent the paternal genetic markers. Blue lines, dotted lines and arrow heads represent the maternal genetic markers. Orange and blue boxes represent the paternal and maternal (GT)_n_ motif 1, respectively. Gray boxes represent the contracted or expanded (GT)_n_ motif 1. **b** Proportion of different clones in heterozygous embryos at different developmental stages. **c** Proportion of different clones in different tissues of an adult heterozygous individual. **d** Proportion of different clones in different adult heterozygous individuals. Numbers of sequenced clones are indicated at the top. M1-M5, the number of male samples. F1-F5, the number of female samples. PC + MC, mixed sample with 4-day embryos of homozygous paternal and maternal control in equal amounts

Next, we examined the linkage patterns of the paternal and maternal makers flanking (GT)_n_ motif 1 in the heterozygous embryos at different developmental stages. More than 20 clones of PCR products amplified from each testing embryonic sample were sequenced and analyzed (Fig. 2a, b). Wild type clones identical to paternal or maternal sequences, intra-allelic recombination clones with contraction or expansion of (GT)_n_ motif 1, and HR clones with linked paternal and maternal genetic markers were detected in all the examined embryos. In the equivalent mixture of 4-day old embryos of homozygous CGF and homozygous JGF, only wild type clone identical to parental alleles and intra-allelic recombination clones with contraction or expansion of (GT)_n_ motif 1 were detected. These results indicate that the HR clones were unlikely to be the products generated by template-switching during PCR reaction. The intra-allelic recombination clones were considered to be generated by template-switching in the PCR reactions and were eliminated in the statistical data.

In some of the HR clones, the paternal-derived sequence was at the upstream and the maternal-derived sequence was at the downstream of the (GT)_n_ motif 1. In the rest of the HR clones, the order of paternal- and maternal-derived sequences was just the opposite (Fig. 2a). These linkage patterns of paternal and maternal makers in the HR clones indicate that reciprocal crossover between the homologous chromosomes occurs around (GT)_n_ motif1. Surprisingly, the proportion of HR clones gradually increased along with embryogenesis and reached to as high as 33.33% in 4-day old embryos (Fig. 2b).

We further examined the frequency of HR around (GT)_n_ motif 1 in various tissues and different individuals of the heterozygote. Frequent mitotic HR was detected in all the examined tissues of an adult heterozygous individual but the frequency was different in each of the examined tissues (Fig. 2c). Frequent mitotic HR was also detected in the genome of caudal fin of all examined adult male and female heterozygous individuals, and the frequency was somewhat different among individuals (Fig. 2d). Taken together, these results demonstrate that HR occurs frequently at (GT)_n_ motif 1 during mitosis of normal somatic cells in vivo.

### HR occurs frequently at innate (GT)_n_ motif 1 in germ cells

**Fig. 3:**
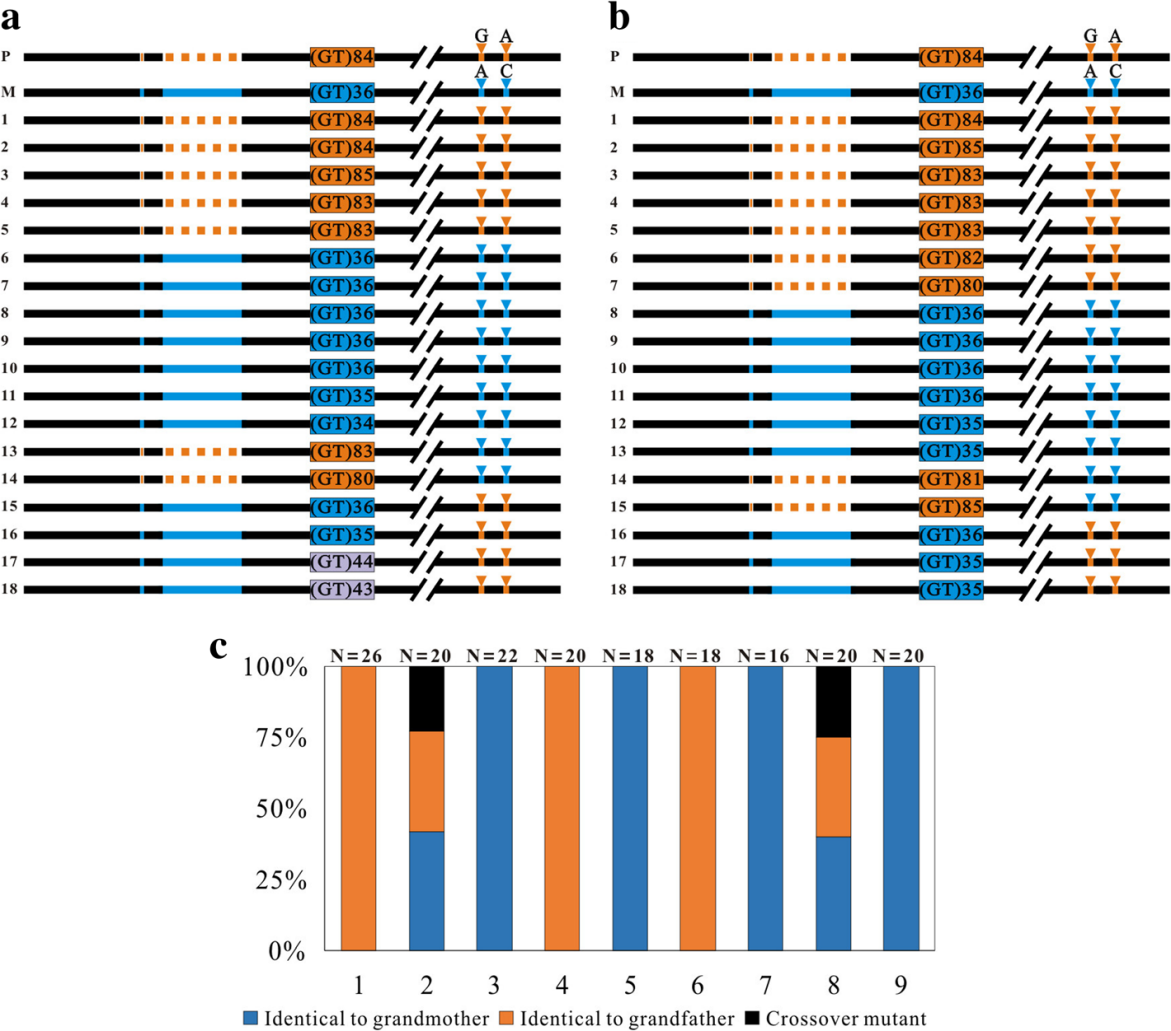
Homologous recombination at the (GT)_n_ motif1 region in germ cells. **a**, **b** The linkage patterns of the paternal and maternal genetic makers flanking the (GT)_n_ motif 1 in the sperm (a) and egg (b) of the heterozygote. Black lines represent the conserved regions. Orange lines, dotted lines and arrow heads represent the paternal genetic markers. Blue lines, dotted lines and arrow heads represent the maternal genetic markers. Orange and blue boxes represent the paternal and maternal (GT)_n_ motif 1, respectively. Gray boxes represent the contracted or expanded (GT)_n_ motif 1. **c** Genotypes in artificial meio-gynogenetic diploid progeny of a male heterozygote individual. 1-9, the numbers of artificial meio-gynogenetic diploid individuals. Number of sequenced clones is indicated at the top

We further examined HR at the (GT)_n_ motif 1 in sperms and eggs of the heterozygote by analyzing the linkage patterns of the paternal and maternal makers flanking the (GT)_n_ motif 1. The results showed that the frequency of HR at the (GT)_n_ motif 1 in sperms and eggs was almost at the same level. About 33.33 and 27.78% of the sequenced clones in the sperm and eggs, respectively, of the heterozygote were HR clones (Fig. 3a, b). Interestingly, the overall proportion of HR clones in the germ cells was almost identical to that in the 4-day old embryonic cells.

Since the frequencies of HR at the innate (GT)_n_ motif 1 were surprisingly high in both somatic and germ cells, we next examined the reliability of the HR frequency by analyzing the genotypes of the progeny from the heterozygote. Considering that, in backcross progeny, frequent HR would occur at (GT)_n_ motif 1 between the wild type CGF and JGF homologous chromosomes derived from the hybrid and a parent of the hybrid, the true HR frequency at (GT)_n_ motif 1 in the germ cells of hybrid could not be obtained by backcross genetic testing. To avoid confusing the HR which occurred in offspring and the HR which occurred in the parent, we used gynogenetic diploid progeny for genotype analysis. Gynogenetic diploid progeny was generated by inducing the development of matured eggs from a female heterozygote with genetic inactivated common carp sperm and then inhibiting the second polar body release of the activated eggs. In this artificial meio-gynogenetic diploid individual, the two alleles in a given locus were the duplicates derived from the same homologous chromosome. The proportion of heterozygous individuals in the gynogenetic diploid progeny of a heterozygote individual directly reflects the true frequency of meiotic HR during oogenesis. By analyzing the linkage pattern of genetic markers flanking the (GT)_n_ motif 1 in the gynogenetic diploid progeny, we observed that 22.22% of the examined gynogenetic diploid individuals (*n* = 9) are heterozygotes. In these gynogenetic diploid individuals, three linkage patterns of genetic markers identical to that in the grandfather, grandmother or crossover homologs were detected (Fig. 3c). 77.78% of the examined gynogenetic individuals were homozygote identical to either grandfather or grandmother (Fig. 3c). The proportion of heterozygous individuals in the gynogenetic diploid progeny of a female heterozygoues was almost equal to proportion of meiotic HR clones detected in the eggs of heterozygote. This confirms that the high frequency of HR at the (GT)_n_ motif 1 in the eggs of heterozygote is indeed reliable.

### HR occurs independently in adjacent regions containing GT repeats in both somatic and germ cells

**Fig. 4:**
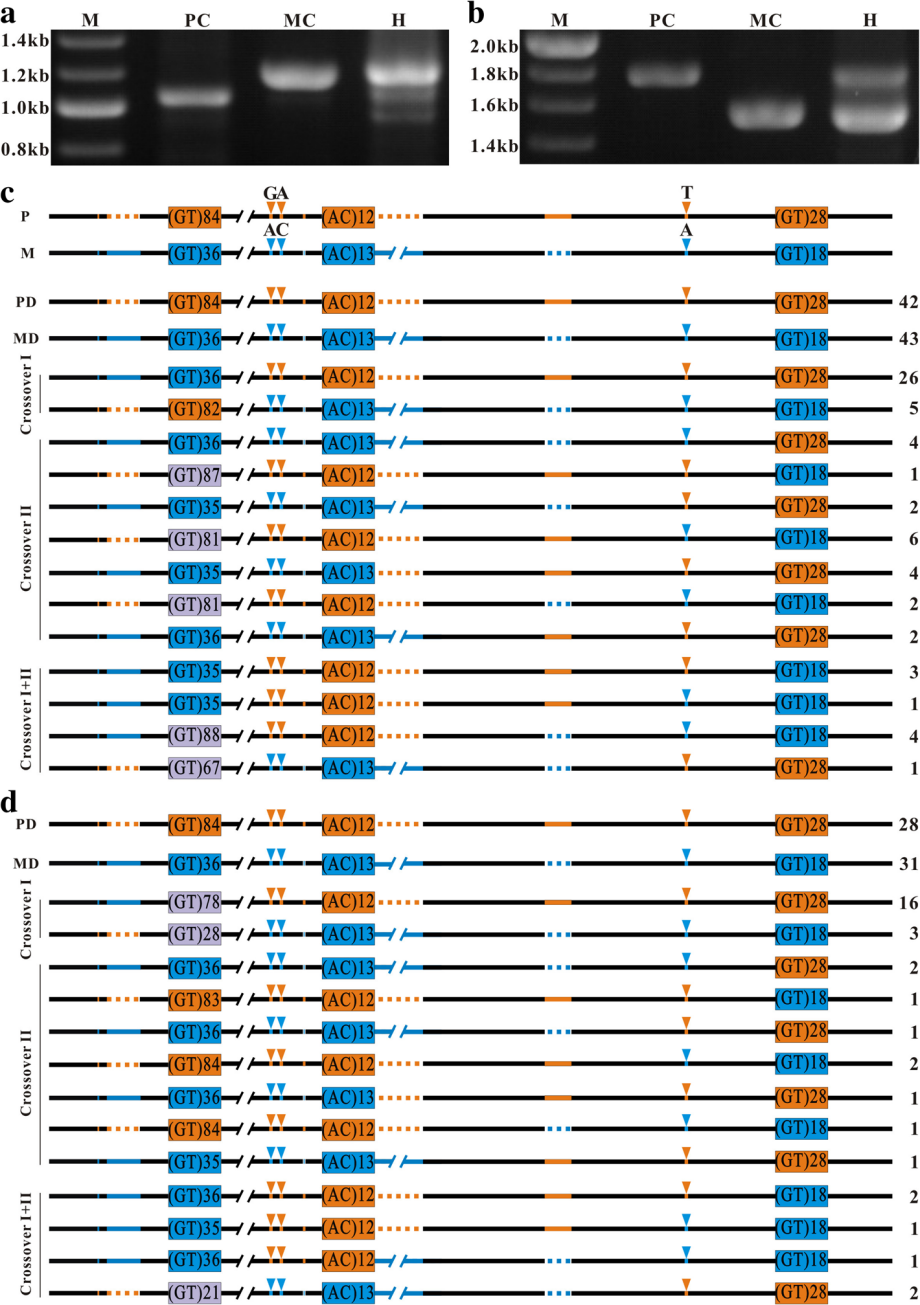
Independent HR events in the adjacent regions containing GT repeats in somatic and germ cells. **a** Gel electrophoretogram of PCR products amplified with a pair of primers spanning the distal *ntl* promoter region containing (GT)_n_ motifs. **b** Gel electrophoretogram of PCR products amplified with a pair of primers spanning the proximal *ntl* promoter region lacking (GT)_n_ motif. M, marker. PC, paternal control. MC, maternal control. H, 4-day old heterozygous embryos. **c**, **d** The linkage patterns of the paternal and maternal genetic makers in the flanking sequences of the three (GT)_n_ motifs in the 4-day old embryos and in the sperm of heterozygote, respectively. Black lines represent the conserved regions. Orange lines, dotted lines and arrow heads represent the paternal genetic markers. Blue lines, dotted lines and arrow heads represent the maternal genetic markers. Orange and blue boxes represent the paternal and maternal (GT)_n_ motif 1, respectively. Gray boxes represent the contracted or expanded (GT)_n_ motif 1. PD, paternal-derived wild type clones. MD, maternal-derived wild type clones. Crossovers I, II, I + II, crossover types. Numbers of the clones are indicated at the right. Note that the exchanged fragments in the co-occurrence of crossovers I and II are identical to the sequence regions from one of the crossover I sites to one of the crossover II sites

If the (GT)_n_ motif 1 induces HR during mitosis and meiosis, one would expect that (GT)_n_ motif 2 and 3 should also promote mitotic and meiotic HR, and no HR occurs at the adjacent *ntl* promoter region lacking GT repeats. Thus, we examined HR events in the distal *ntl* promoter region containing the three (GT)_n_ motifs and the proximal *ntl* promoter region (from the (GT)_n_ motif 3 to the translation start site) lacking GT repeats. In the genome of 4 day-older embryos, PCR products different from the parental-derived products in length were detected in the distal *ntl* promoter region (Fig. 4a), but not in the proximal region (Fig. 4b). Sequencing analysis further confirmed that HR occurs in the distal promoter region containing the three (GT)_n_ motifs (Fig. 4c), but not in the proximal promote region lacking (GT)_n_ motifs (data not shown).

Of the 146 sequenced clones amplified from the distal region containing the three (GT)_n_ motifs, the percentage of paternal-derived (PD) and maternal-derived (MD) wild type clones was 58.22% (*n* = 42 + 43), and the percentage of HR clones was 41.78% (*N* = 61, Fig. 4c). The linkage patterns of paternal and maternal makers in the HR clones showed that two types of independent reciprocal crossover events, named crossover I and II, and one short fragment exchange event occurred in the region containing the three (GT)_n_ motifs (Fig. 4c). Crossover I occurred around the (GT)_n_ motif 1 as described above (Fig. 4c; Additional file 2: Figure S4). Crossover II occurred between the motif 2 and 3 at different locations (Fig. 4c; Additional file 2: Figure S5). The percentage of crossover I clones and crossover II clones was 21.23% (*n* = 31) and 14.39% (*n* = 21), respectively. In 6.16% of HR clones (*n* = 9), only a short fragment was exchanged between the paternal and maternal homologous chromosomes (Fig. 4c; Additional file 2: Figure S6), and they appeared as a non-crossover recombination event. However, the exchanged fragments were identical to the sequence regions from one of the crossover I sites to one of the crossover II sites, indicating that the exchanged fragments were produced by co-occurrence of the crossover I and II in the adjacent regions.

In germ cells, HR also occurred independently in the adjacent regions containing GT repeats in a similar manner. Independent crossover event I and II were also detected exactly at the same sites in the genome of sperm (Fig. 4d). The proportions of the three types of clones in the sperms were almost equal to that as observed in the 4-day old embryonic cells. Of the 93 sequenced clones, the proportion of paternal- and maternal-derived clones was 63.44% (*n* = 23 + 31), the proportion of crossover I clones, II clones and co-occurring crossover clones were 20.43% (*n* = 19), 9.68% (*n* = 9) and 6.45% (*n* = 6), respectively.

### A longer GT repeat tract is more active than a shorter one in promoting DSBs and triggering HR in both somatic and germ cells

**Fig. 5:**
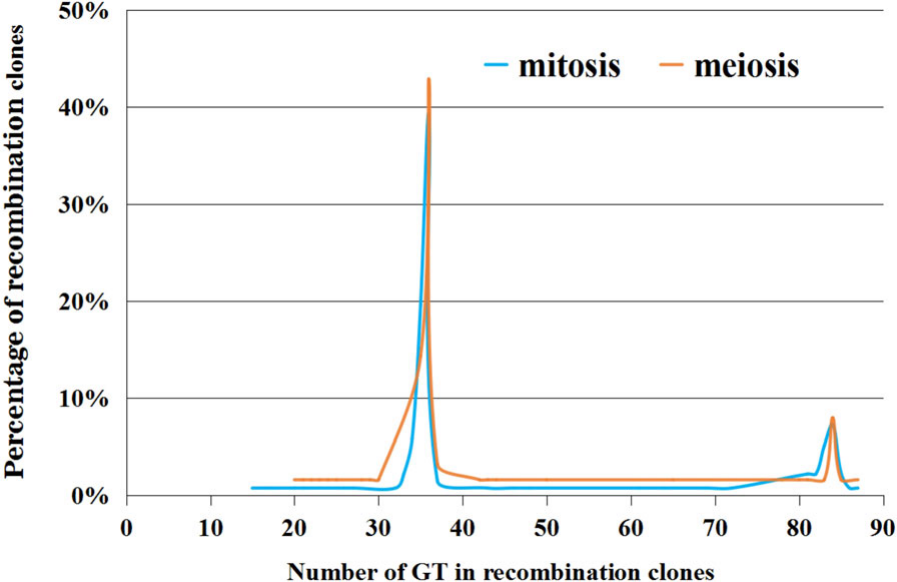
The distribution of HR clones containing different number of GT units in somatic and germ cells

Since paternal (GT)_n_ motif 1 is much longer than the maternal one, the paternal or maternal-derived (GT)_n_ motif 1 in the HR clones can inform us how the HR at (GT)_n_ motif 1 is carried out. To determine whether the length of innate GT repeat tract has an effect on promoting HR, the length of (GT)_n_ motif 1 in all the sequenced HR clones from the somatic cells was analyzed. The percentage of HR clones containing different number of GT units in (GT)_n_ motif 1 is shown in Fig. 5 according to their cell origin.

In 58.10% of mitotic HR clones (81/138), the number of GT units in (GT)_n_ motif 1 is equal to that of maternal (GT)_n_ motif 1. In 12.32% of mitotic HR clones (17/138), the number of GT units in (GT)_n_ motif 1 is equal to that of paternal (GT)_n_ motif1. The remainder of the mitotic HR clones contain (GT)_n_ motif 1 with different contraction or expansion. The percentages of these mitotic HR clone are very low and almost at the same level. These results indicated that 58. 10% of the mitotic HR clones was induced by DSB in the paternal derived chromosome and repaired by using the maternal derived chromosome template; and 12.32% of the mitotic HR clones was induced by DSB in the maternal derived chromosome and repaired by using the paternal derived chromosome template.

The percentage of HR clones containing different number of GT units in (GT)_n_ motif from the germ cells was similar to that observed in the somatic cells. The number of HR clones containing short maternal (GT)_n_ motif 1 was much larger than that of HR clones containing long paternal (GT)_n_ motif 1 (Fig. 5). Of the 63 sequenced HR clones from germ cells, 57.10% of the HR clones (*n* = 36) was induced by DSBs in the paternal derived chromosome and repaired by the maternal derived chromosome template, 9.52% of the HR clones (*n* = 6) was induced by DSB in the maternal derived chromosome and repaired by the paternal derived chromosome template. Therefore, the long (GT)_n_ motif 1 is more susceptible to DSBs than the short one in both somatic and germ cells.

## Discussion

In this study, we used an in vivo system to investigate whether spontaneous DNA damage at GT microsatellites during genome replication has biological importance in promoting HR in vertebrate somatic and germ cells. By examining the linkage pattern of multiple paternal and maternal markers flanking innate GT microsatellites, we analyzed HR at the GT microsatellites in different somatic cells and germ cells in the intraspecific heterozygote. In the embryos, the HR products accumulate gradually as development proceeds. The frequency of HR at the GT microsatellites in normal advanced embryos, adult tissues and germ cells of the heterozygote is surprisingly high, and the type of exchanges between the homologous chromosomes is similar in normal advanced embryos and germ cells. Furthermore, a long GT microsatellite is more active than a short one in promoting HR in both somatic and germ cells.

### The HR products detected in the germ cells may be a combination of mitotic and meiotic HR products

The gradually increases proportion of HR clones during development suggests that the incidence of HR at the (GT)_n_ motifs correlates with the number of cell divisions. The variation of HR frequency observed among tissues also can be explained by the number of stem cell divisions during the development of the tissues. These phenomena are consistent with the characteristics of microsatellites prone to DSBs and inducing repair during genome replication in the process of cell division. Therefore, we infer that mitotic HR at the (GT)_n_ motifs is initiated by genome replication.

HR occurs at the (GT)_n_ motifs in the germ cells in a similar rate and produces similar recovered clone types as in the 4-day old embryos, strongly suggesting that HR at the (GT)_n_ motifs in germ cells is also initiated by genome replication. During gametogenesis, HR induced by genome replication errors, stalling and slippage at (GT)_n_ motifs can occur during mitosis of germ cell precursors at proliferative stage and during meiosis of germ cells. Thereby, the HR products detected at the (GT)_n_ motifs in germ cells may be a combination of mitotic HR generated during proliferation of germ cell precursors and meiotic HR generated during meiosis of germ cells.

### Frequent HR at innate (GT)_n_ motifs may be beneficial to maintain genomic stability and suppress LOH

During DSBs repair, sister chromatids are held in proximity by cohesion whereas the homologous chromosomes may be more distant from each other, so that intact sister chromatid is readily selected as the repair template [3]. More importantly, it has been demonstrated that RecQ helicase BLM can actively suppress crossover between homologous chromosomes, which can elicit detrimental LOH of the entire chromosome arm that is distal to the HR event, by dissolving recombination intermediates containing a double Holliday junction [26–28]. Therefore, mitotic HR is in general an infrequent outcome of DSBs repair [3], and the high frequency of HR detected at (GT)_n_ motifs in somatic tissues is very surprising. This unusual phenomenon can be explained by the frequency of replication stalling and because slippage at (GT)_n_ motifs is so high that DSBs occurs simultaneously at the same position on the two sister chromatids during normal mitosis. In this condition, no sister chromatid can be employed as the repair template by the DSBs repairing apparatus. Thereby, frequent HR at (GT)_n_ motifs is an essential error correction procedure of normal mitosis for maintaining genomic stability during genome replication.

**Fig. 6:**
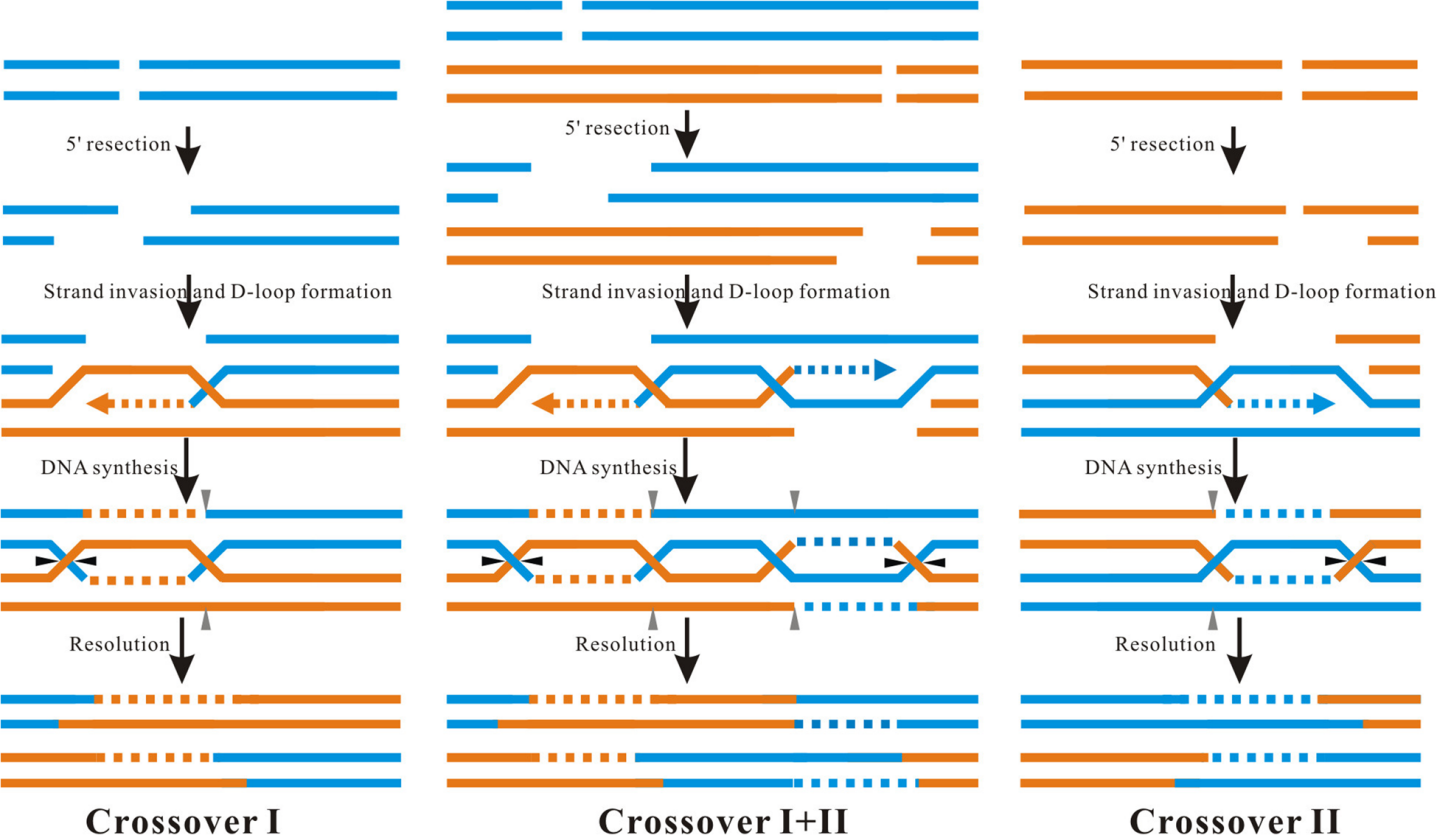
Co-occurrence of crossovers in adjacent regions can result in non-crossover recombination. DSBs are formed in the adjacent regions on each of the homologous chromosomes. HR initiates with end resection at the DSBs sites on each of the homologous chromosomes, which produces a 3′ single-stranded end that can invade a homologous template to initiate repair. The second single-stranded end is captured by the D-loop to form a double Holliday junction. The double Holliday junction can be resolved by strand cleavage and result in a crossover (cleavage at arrowheads on one side) outcome (Crossover I and Crossover II). Two double Holliday junctions formed in the adjacent region will result in a four strand recombinant structure. The co-occurrence of crossovers in the adjacent regions would dissolve the Holliday junction of the adjacent DSBs repair intermediates and result in short fragment exchange between the homologous chromosomes (Crossover I + II), looking like a non-crossover recombination event. Arrowheads indicate the cleavage sites

A previous study has observed that, in a yeast chromosome, insertion of a GT repeat close to a meiotic HR hotspot can inhibit the progression of the strand exchange during meiosis by stimulating two double crossovers [20]. Our study showed that co-occurrence of independent crossovers in the adjacent regions containing GT repeats results in short segment exchange between the homologous chromosomes in both somatic and germ cells (Fig. 4c, d). It is likely that frequent replication stalling and slippage at (GT)_n_ motifs induces simultaneously DSBs in the adjacent GT repetitive regions on each of the homologous chromosomes. DSBs initiate 5′-3′ resection of the ends. Subsequently, the 3′ overhang invades into the homologous template DNA and primes DNA synthesis to form a structure called D-loop and results in a double Holliday junction. Two double Holliday junctions formed in the adjacent region would form a four strand recombinant structure. In this case, the co-occurrence of crossovers in the adjacent GT repetitive regions would dissolve the Holliday junction of the adjacent DSBs repair intermediates and result in short segment exchange between the homologous chromosomes (Fig. 6). Therefore, frequent HR at adjacent (GT)_n_ motifs in a chromosome would be beneficial to suppress LOH of a long chromosomal region encompassing multiple genetic loci during mitotic HR.

### Frequent DSB repairing at (GT)_n_ motifs may result in frequent bivalent formation during mitosis and play a role in preleptotene pairing during meiosis

The high frequency of mitotic HR at (GT)_n_ motifs means that the homologous chromosomes are frequently held together in a bivalent structure by crossover during normal mitosis. The eukaryotic recombinase Rad51 orthologs, which have high affinity for GT repeat sequence [21, 22], may play an important role in initiating mitotic bivalent formation and inhibit non-homologous end joining pathway of DSB repairing during normal mitosis. Because eukaryotic organisms contain many (GT)_n_ motifs in their genomes [12–14], replication errors and stalling may initiate crossovers at different GT repeat loci and hold the homologous chromosomes together in different bivalents during normal mitosis in eukaryotes. Reasonably, frequent mitotic bivalent formation mediated by (GT)_n_ motifs would be beneficial to preventing HR between non-allelic genomic fragments that share high sequence similarity but are at different locations on the same chromosome or on different chromosomes.

DSBs formation by the ortholog of Spo11, an evolutionally conserved type II topoisomerase-like protein, appears to be a universal feature of meiotic pairing in eukaryotic organisms [2]. However, it has been reported that in mice a significant proportion of homolog pairing is established prior to the introduction of programmed DSBs catalyzed by SPO11, and the DNA cleavage activity of SPO11 is not required for homolog pairing during meiosis [29]. How homologous chromosomes find each other and initiate the pairing process during meiosis remains to be understood. In consideration of that, there are many (GT)_n_ motifs scattered in the eukaryotic genome. Frequent DSBs formation at GT repeats in pre-meiotic S-phase may play an important role in preleptotene pairing of homologous chromosomes during normal meiosis.

### LOH at *ntl* locus during embryogenesis might have a selective advantage for heterozygote

In general, LOH in somatic cells is harmful and associated with many abnormalities in mammals [1, 3, 4]. We observed that two independent mitotic HR events at (GT)_n_ motifs led to frequent LOH at the nearby *ntl* locus in somatic cells during normal embryogenesis. This result implicates that a significant fraction of embryonic cells in a heterozygous individual could be converted from heterozygous to homozygous state at *ntl* locus during development. Gavin that (GT)_n_ motif 1 in the promoter of *ntl* is conserved in different species of fish. LOH at *ntl* locus mediated by (GT)_n_ motif 1 during embryogenesis may have a biological role. Tischfield has proposed that in vertebrates LOH at those loci whose proper action depends on the concentration of its gene products might have a selective advantage for the heterozygote, such as correcting mutant phenotype or promoting homozygosity of normal alleles [4]. Indeed, high concentration of Ntl/Brachyury is required for dorsal mesoderm formation in the vertebrates and haplo-insufficiency has been detected in mammals but not in fish [30].

## Conclusions

HR occurs frequently at innate GT microsatellites in a similar length-dependent manner in normal somatic and germ cells, and the incidence of HR is correlated with the number of cell divisions. Therefore, HR products detected at GT microsatellites in germ cells may include mitotic HR products generated during mitosis in germ cell precursors and meiotic HR products generated during meiosis in germ cells. Since there are many scattered (GT)_n_ motifs in the eukaryotic genome, it would be interesting to investigate whether adjacent (GT)_n_ motifs on the same chromosome could suppress extensive strand exchange during normal mitosis and meiosis, and whether GT microsatellites is an essential genome element for meiotic bivalent formation.

## Methods

### Animals

The Chinese goldfish (CGF) strain *C. auratus auratus* and the Japanese goldfish (JGF) strain *C. auratus cuvieri*, were purchased from different goldfish bred farms and maintained in our laboratory in the breeding season according to the Guidelines for the Care and Use of Laboratory Animals of Zhejiang University. The intraspecific heterozygote of goldfish was generated by crossing with a homozygous CGF male individual and a homozygous JGF female individual. Gynogenetic diploid progeny of the heterozygote was generated as described previously [31]. The matured eggs from a female heterozygote were activated by genetic inactivated common carp sperm. Two minutes after activation, the activated eggs were cold shocked at 0-2 °C for 15 min to inhibit the second polar body release. All experimental protocols were approved by the Ethics Committee of Laboratory Animal Center of Zhejiang University (Approval no. Zju 201,306-1-11-060).

### Samples preparation

Heterozygous embryos were maintained at 22 °C and collected at desired stages. Sperms and eggs of the heterozygote were obtained from breeding heterozygous males and females, respectively, by gentle squeezing. To avoid contamination from genomes of somatic cells, the unfertilized eggs were dechlorionated with 0.2% trypsin and then washed three times with water before flash freezing. All the samples were flash frozen in liquid nitrogen until genomic DNA extraction.

### Cloning *ntl* promoter sequence

A 2.6 kb long *ntl* promoter fragment upstream of the ATG was amplified from the genomic DNA of the two goldfish strains by Genome Walker™ Universal Kit (Clontech, USA), separately, and sequenced. The obtained sequences of *ntl* promoter have been submitted to Genbank (Accession numbers for *C. carassius cuvieri:* KU870661; *C. carassius*: KU870662).

### (GT)_n_ motif 1 stability and homologous recombination analysis

The stability of (GT)_n_ motif 1 was tested by specific PCR with a pair of primers: 5’-TATTTTGGTTCGGACAAAGTG-3′(forward); 5’-GATGAGTCAGGGCCTGGAAA-3′ (reverse). HR at different regions of the *ntl* promoter was analyzed by specific PCR and sequence comparing. The sequences of primers used for amplification of the (GT)_n_ motif1 and its flanking sequences are 5’-ACACACTTTGAACTTTCTATTTATTA-3′ (forward) and 5’-GTGTGCTCAGTGCTGGAGGGGTTA-3′ (reverse). PCR reactions was performed at 94 °C for initial denaturation for 5 min, followed by 32 cycles of 94 °C for 30 s, 55 °C for 30s, 72 °C for 1 min, finally elongated for 7 min at 72 °C. The sequences of primers used for amplification of the region covering the three (GT)_n_ motifs and its flanking sequences are 5’-ACACACTTTGAACTTTCTATTTATTA-3′ (forward) and 5′-TTGGAGTTG ATGATAGAGGATGTTGAT-3′ (reverse). The sequences of primers used for amplification of the sequence from the (GT)_n_ motif 3 to the transcription start site are 5’-GCGGGCTGCTCACTTTCTCACTTATAC-3′ (forward) and 5’-CGTCCAGTT TTAGTGACAATCATT-3′ (reverse). PCR reactions was performed at 94 °C for 10 s, followed 5 cycles of 94 °C for 30 s, 55 °C for 30 s, 72 °C for 2 min, and 28 cycles of 94 °C for 30 s, 50 °C for 30 s, 72 °C for 2 min,lastly elongated for 7 min at 72 °C. All PCR master mixes contained (in 50 μL): 1 × Ex-Tag Buffer (Takara, Japan), 250 μM dNTP mix, 200 nM for both primers, 10 ng-100 ng genomic DNA and 2.5 U Ex-Taq DNA polymerase (Takara, Japan).

## Additional files

Additional file 1: Genome sequences of the paternal and maternal *ntl* promoter region containing the (GT)_n_ motifs. **Figure S1.** Alignment of paternal and maternal goldfish distal *ntl* promoter region containing the (GT)_n_ motifs. **Figure S2.** Sequencing spectrums of the paternal distal *ntl* promoter region containing the (GT)_n_ motifs. **Figure S3.** Sequencing spectrums of the maternal distal *ntl* promoter region containing the (GT)_n_ motifs. (DOCX 1620 kb)

Additional file 2: Genome sequences of various reciprocal crossovers clones at different sites of *ntl* promoter. **Figure S4.** Sequencing spectrums of reciprocal crossover I clones in *ntl* promoter. **Figure S5.** Sequencing spectrums of reciprocal crossover II clones in *ntl* promoter. **Figure S6.** Sequencing spectrums of HR clones that only a fragment is exchanged between the paternal and maternal homologous chromosomes. (DOCX 3959 kb)

## Abbreviations

CGF: Chinese goldfish
DSBs: Double-strand breaks
HR: Homologous recombination
JGF: Japanese goldfish
LOH: Loss of heterozygosite
PCR: Polymerase chain reaction
