# Supplementary material for: Homologous recombination occurs frequently at innate GT microsatellites in normal somatic and germ cells in vivo
Source: BMC Genomics. 2018 May 11;19:359. doi: 10.1186/s12864-018-4758-y (PMC5948810; doi:10.1186/s12864-018-4758-y)
Supplement: Supplementary file 1 — Genome sequences of the paternal and maternal ntl promoter region containing the (GT)n motifs. Figure S1. Alignment of paternal and maternal goldfish distal ntl promoter region containing the (GT)n motifs. Figure S2. Sequencing spectrums of the paternal distal ntl promoter region containing the (GT)n motifs. Figure S3. Sequencing spectrums of the maternal distal ntl promoter region containing the (GT)n motifs. (DOCX 1620 kb) [file 12864_2018_4758_MOESM1_ESM.docx]

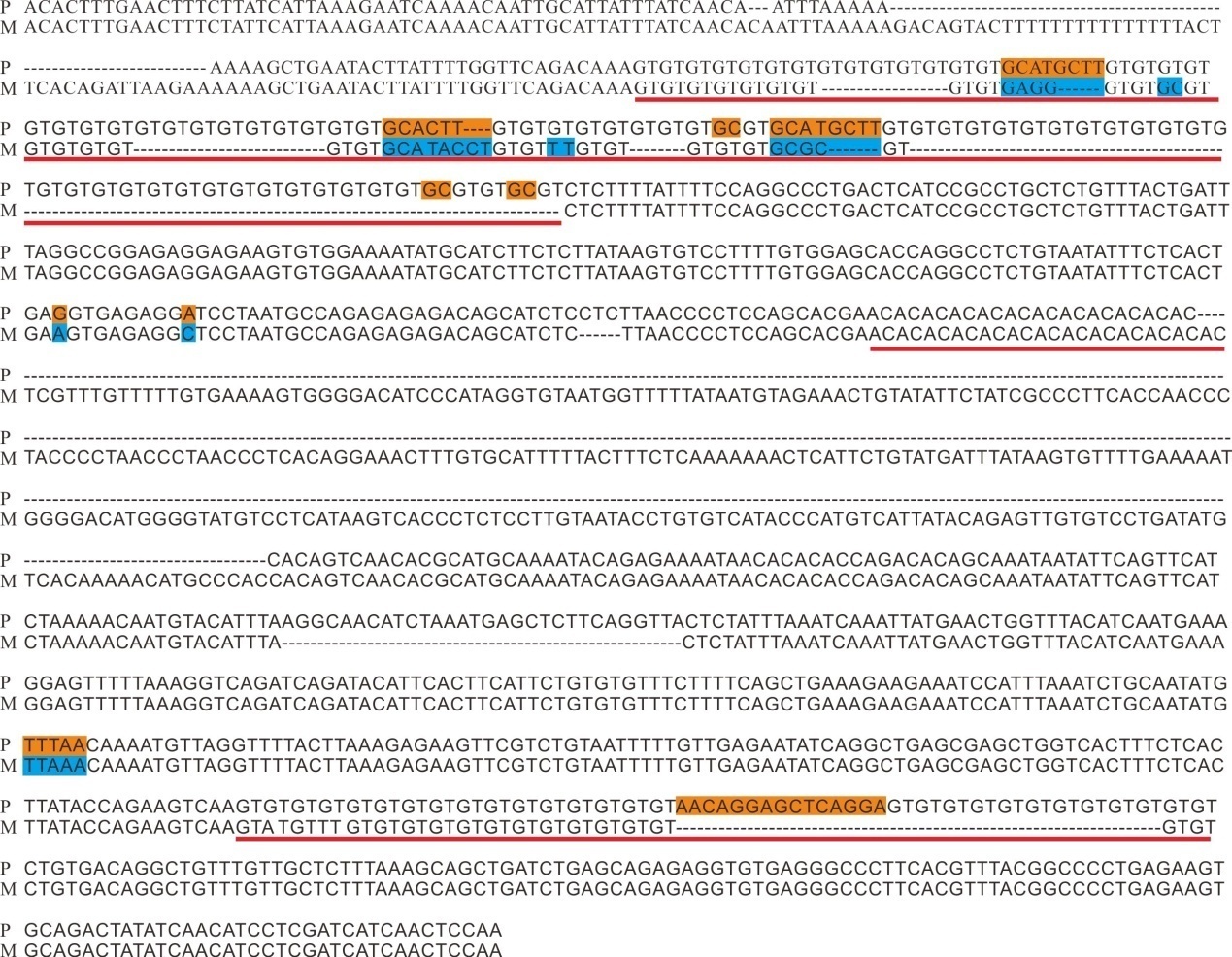


**Figure S1.** Alignment of paternal (P) and maternal (M) goldfish distal *ntl* promoter region containing the (GT)_n_ motifs. Red underlines indicate the (GT)_n_ motifs. Dotted lines represent the deleted sequences. The orange and blue boxes indicate the paternal and maternal genetic markers, respectively.

**
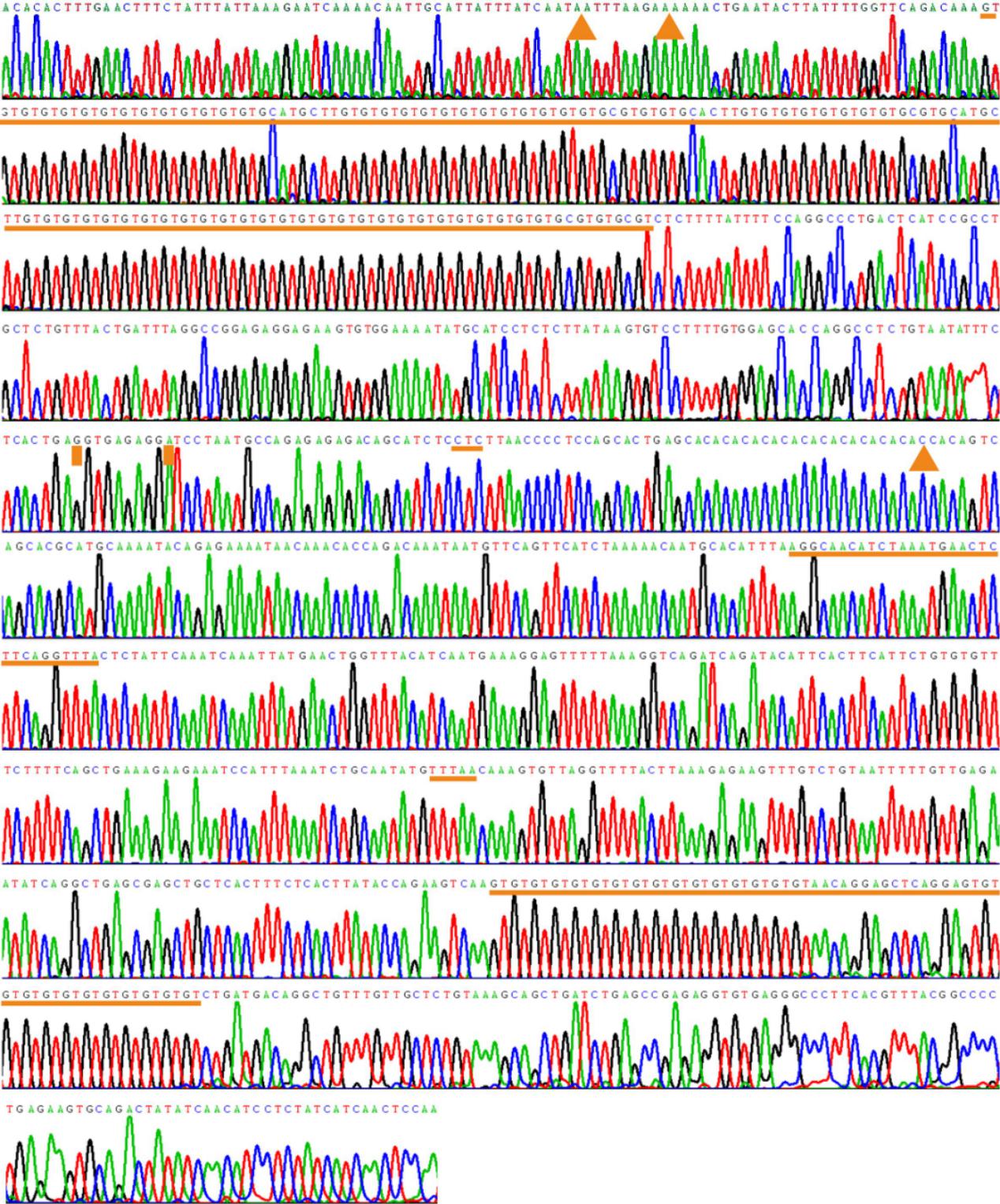
**

**Figure S2.** Sequencing spectrums of the paternal distal *ntl* promoter region containing the (GT)_n_ motifs. The markers are the same as described in Figure S2. Orange arrow head indicate the site a sequence being deleted in paternal genome comparing with maternal genome. Orange underlines indicate the paternal specific sequences. Orange boxes indicate the paternal specific SNP sites.

**
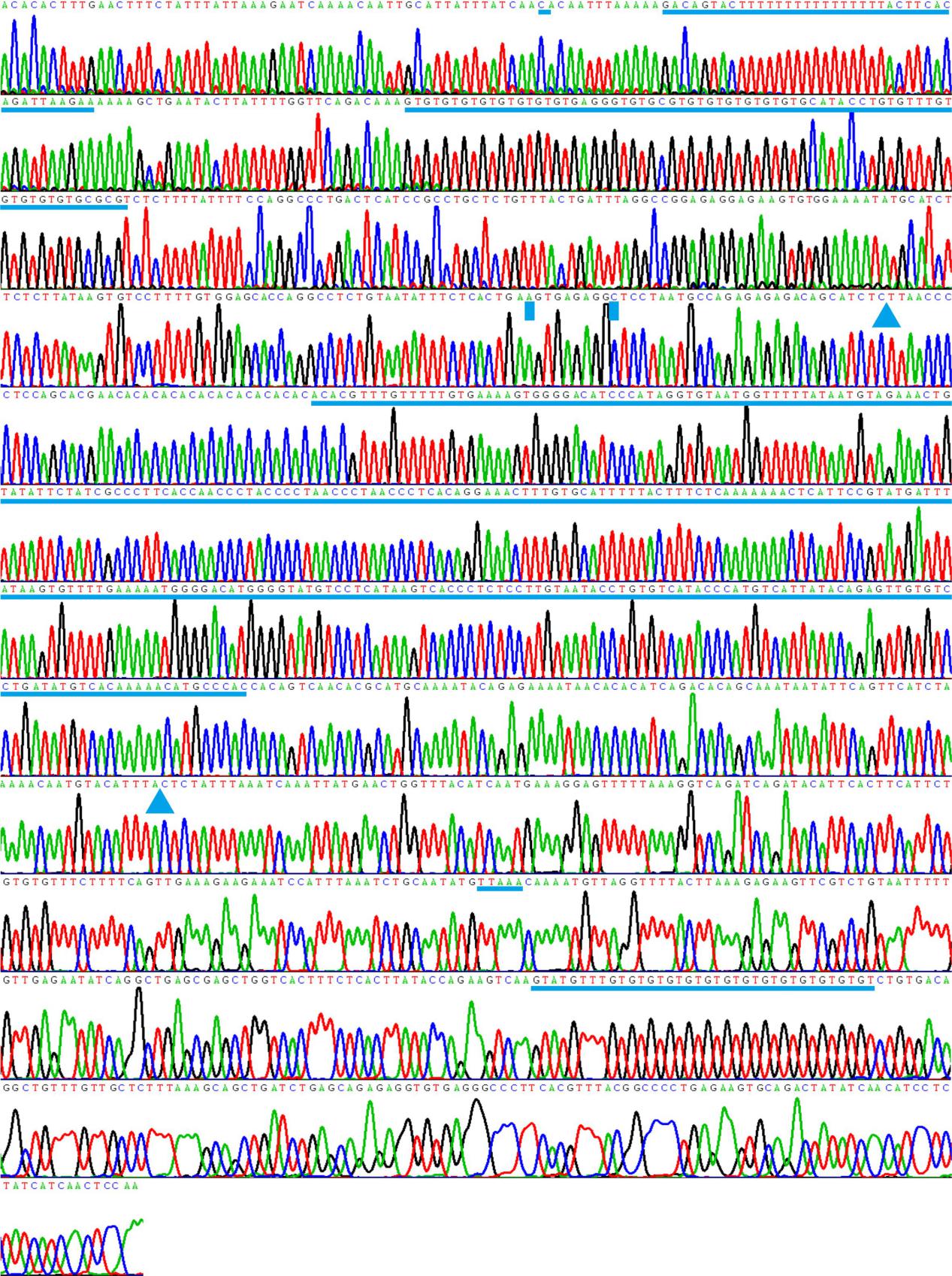
**

**Figure S3.** Sequencing spectrums of the maternal distal *ntl* promoter region containing the (GT)_n_ motifs. Blue arrow head indicate the site a sequence being deleted in maternal genome comparing with paternal genome. Blue underlines indicate the maternal specific sequences. Blue boxes indicate the maternal specific SNP sites.
