# Supplementary material for: Homologous recombination occurs frequently at innate GT microsatellites in normal somatic and germ cells in vivo
Source: BMC Genomics. 2018 May 11;19:359. doi: 10.1186/s12864-018-4758-y (PMC5948810; doi:10.1186/s12864-018-4758-y)
Supplement: Supplementary file 2 — Genome sequences of various reciprocal crossovers clones at different sites of ntl promoter. Figure S4. Sequencing spectrums of reciprocal crossover I clones in ntl promoter. Figure S5. Sequencing spectrums of reciprocal crossover II clones in ntl promoter. Figure S6. Sequencing spectrums of HR clones that only a fragment is exchanged between the paternal and maternal homologous chromosomes. (DOCX 3959 kb) [file 12864_2018_4758_MOESM2_ESM.docx]

a

**
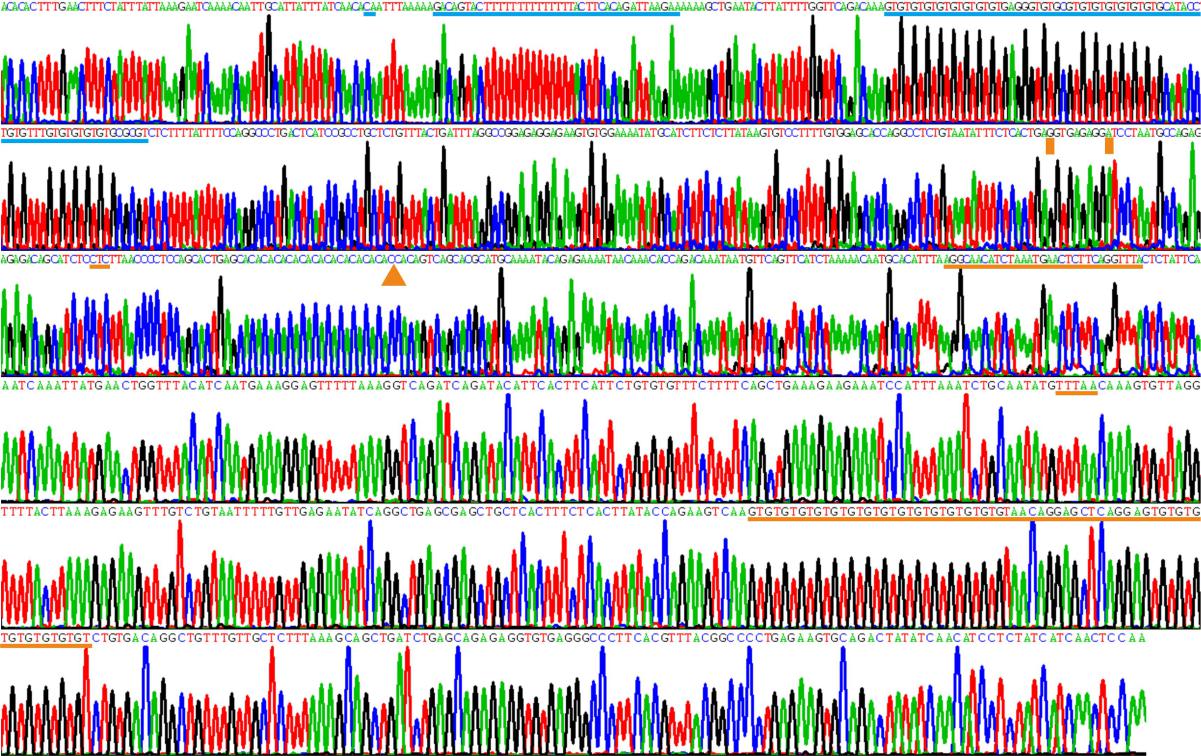
**

**b**

**
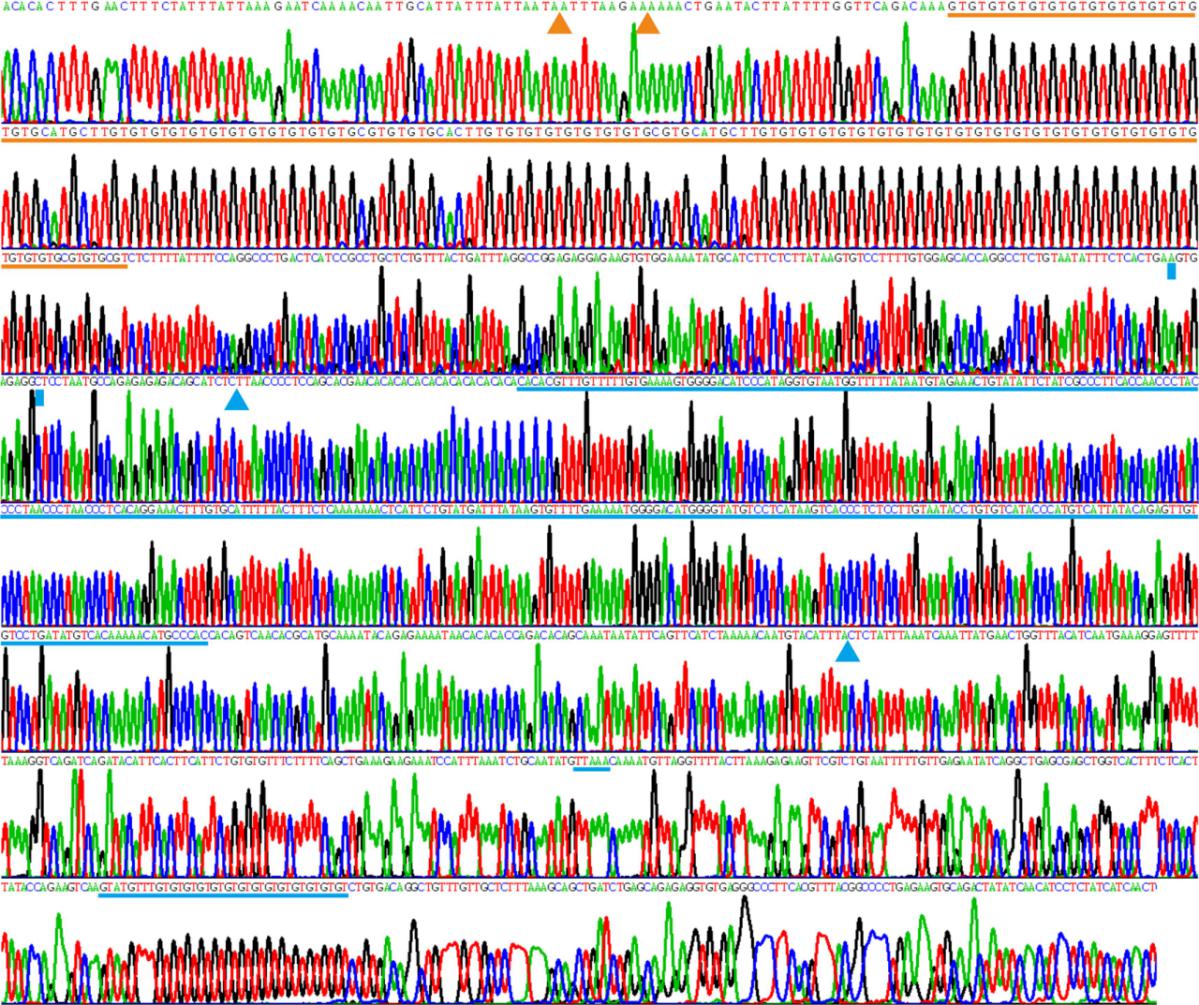
**

**Figure S4.** Sequencing spectrums of reciprocal crossover I around (GT)_n_ motif 1 at *ntl* promoter. Orange and blue arrow heads indicate the site a sequence being deleted in paternal and maternal genomes, respectively. Orange and blue underlines indicate the paternal and maternal specific sequences, respectively. Orange and blue boxes indicate the paternal and maternal specific SNP sites, respectively. **a** and **b** corresponds to the range of crossover I clones, respectively, in Fig. 4c.

**a**


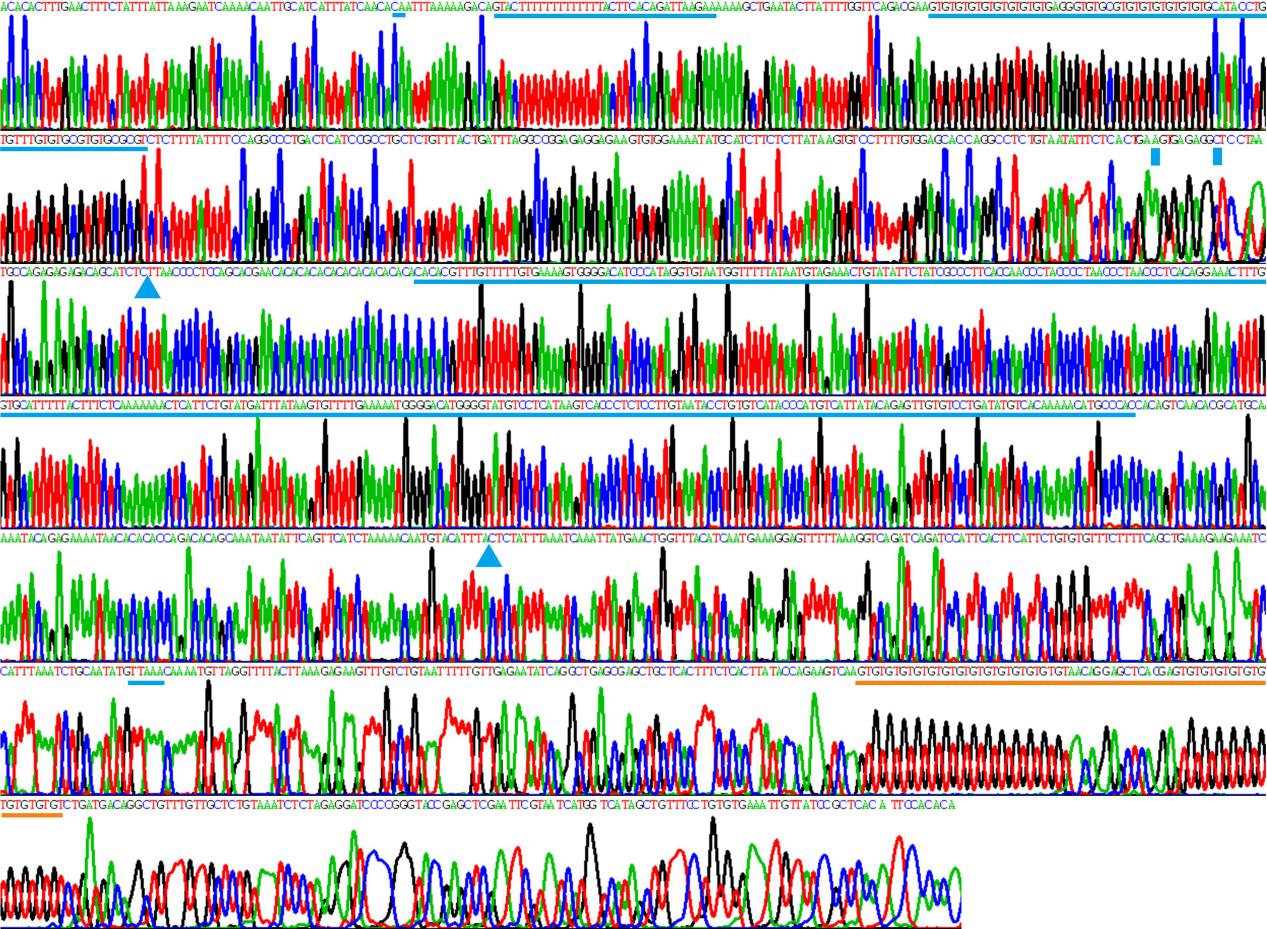


**b**


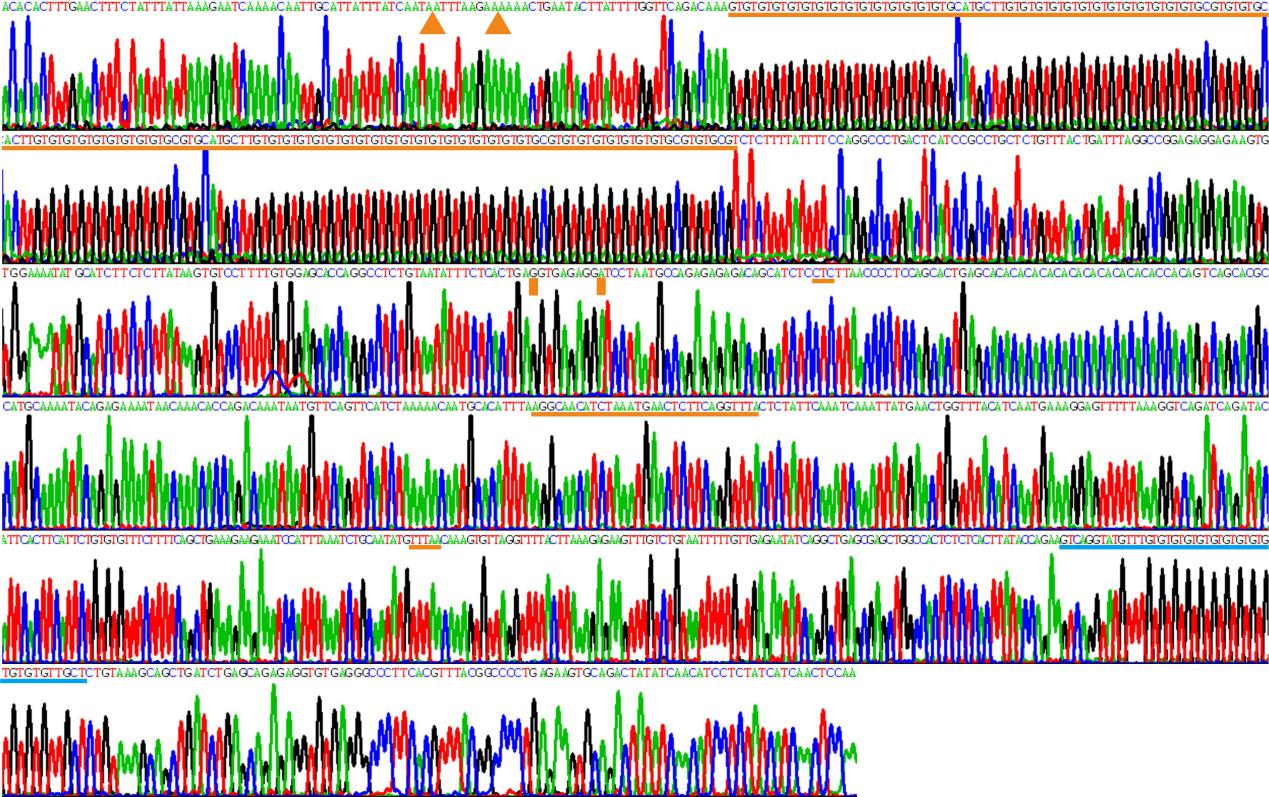


**c**


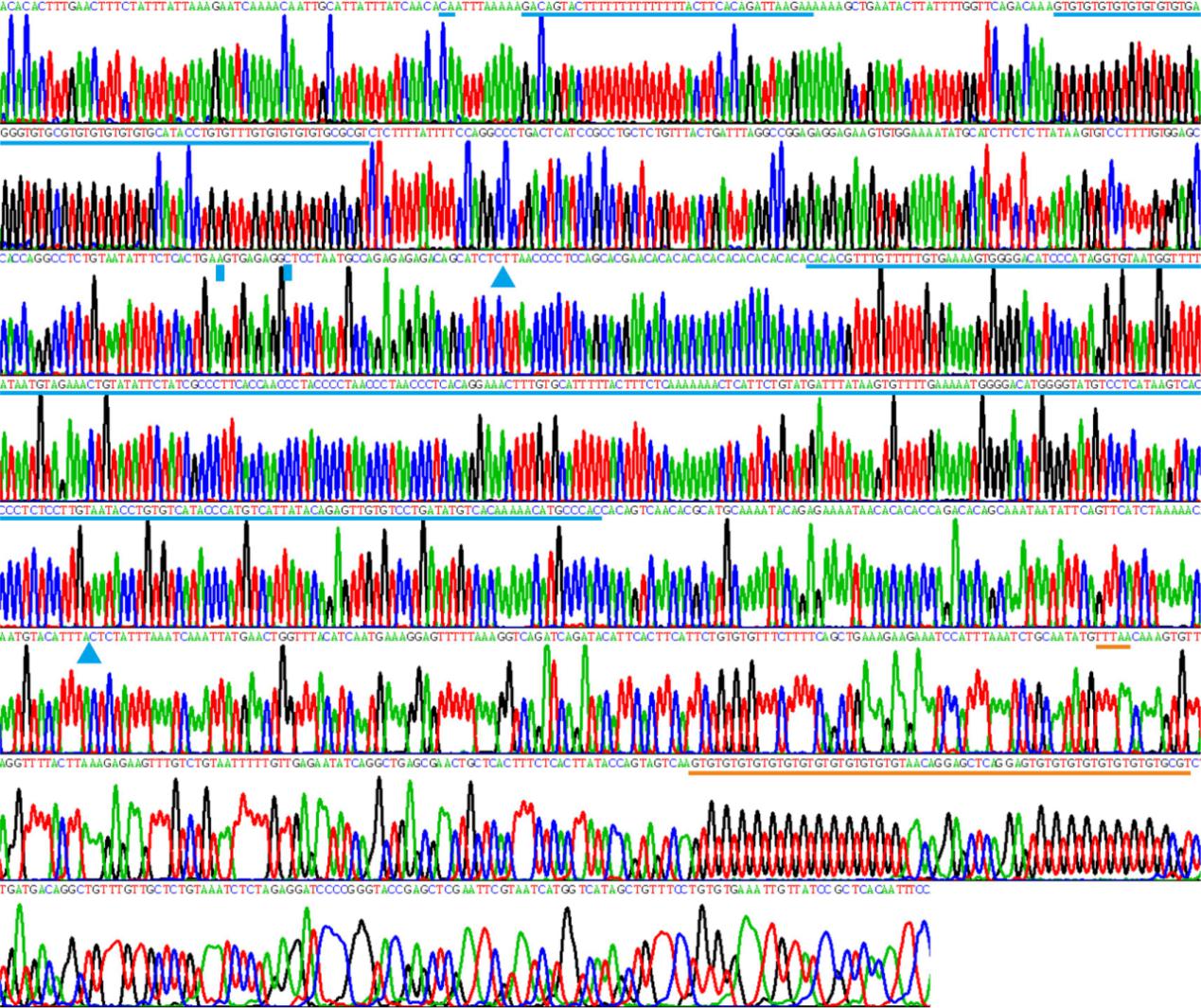


**d**


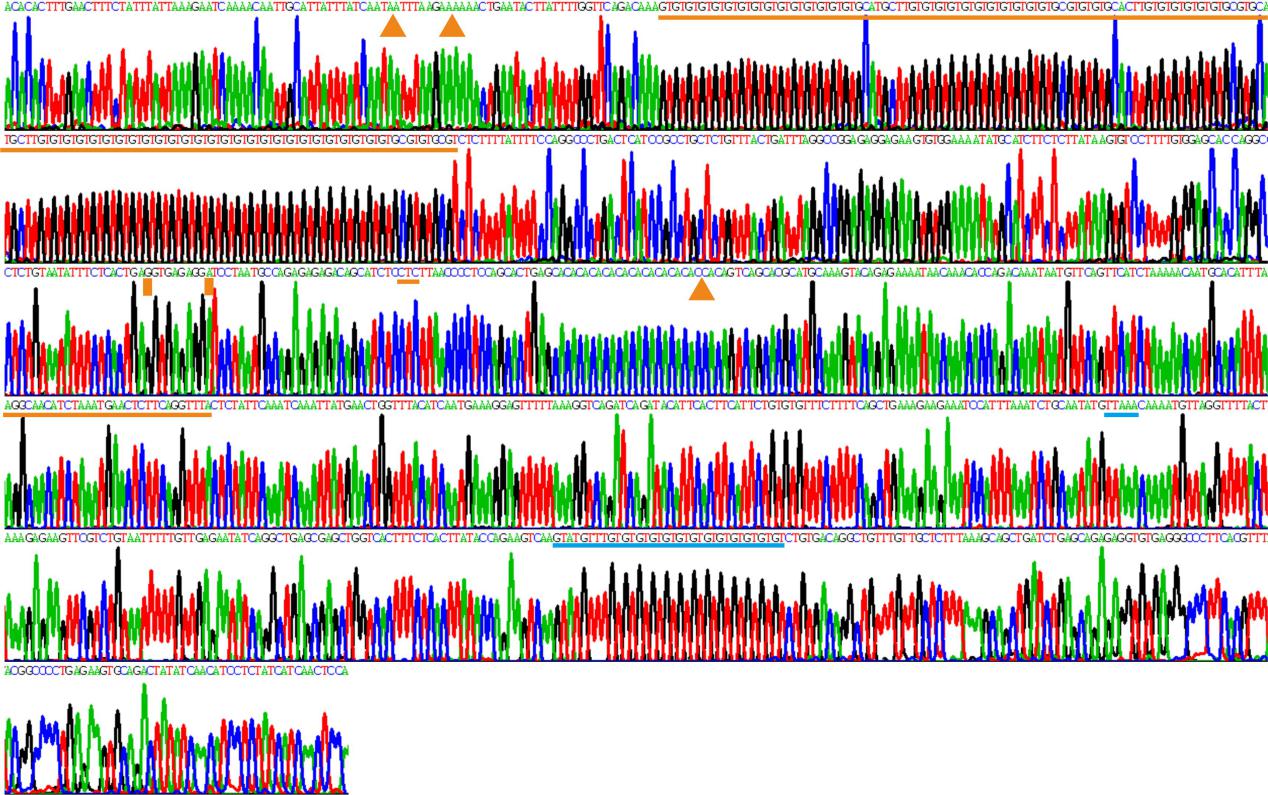


**e**


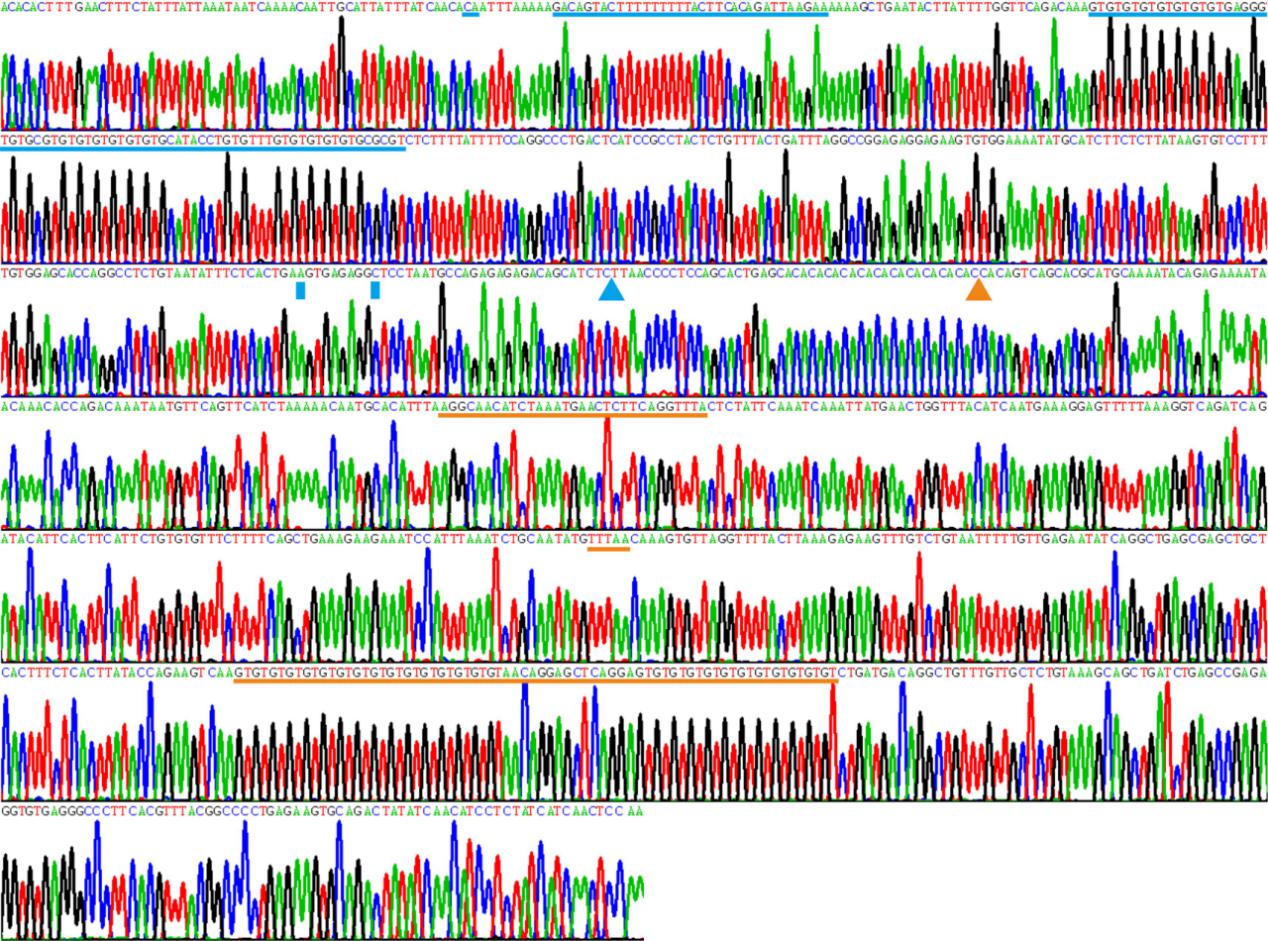


**f**


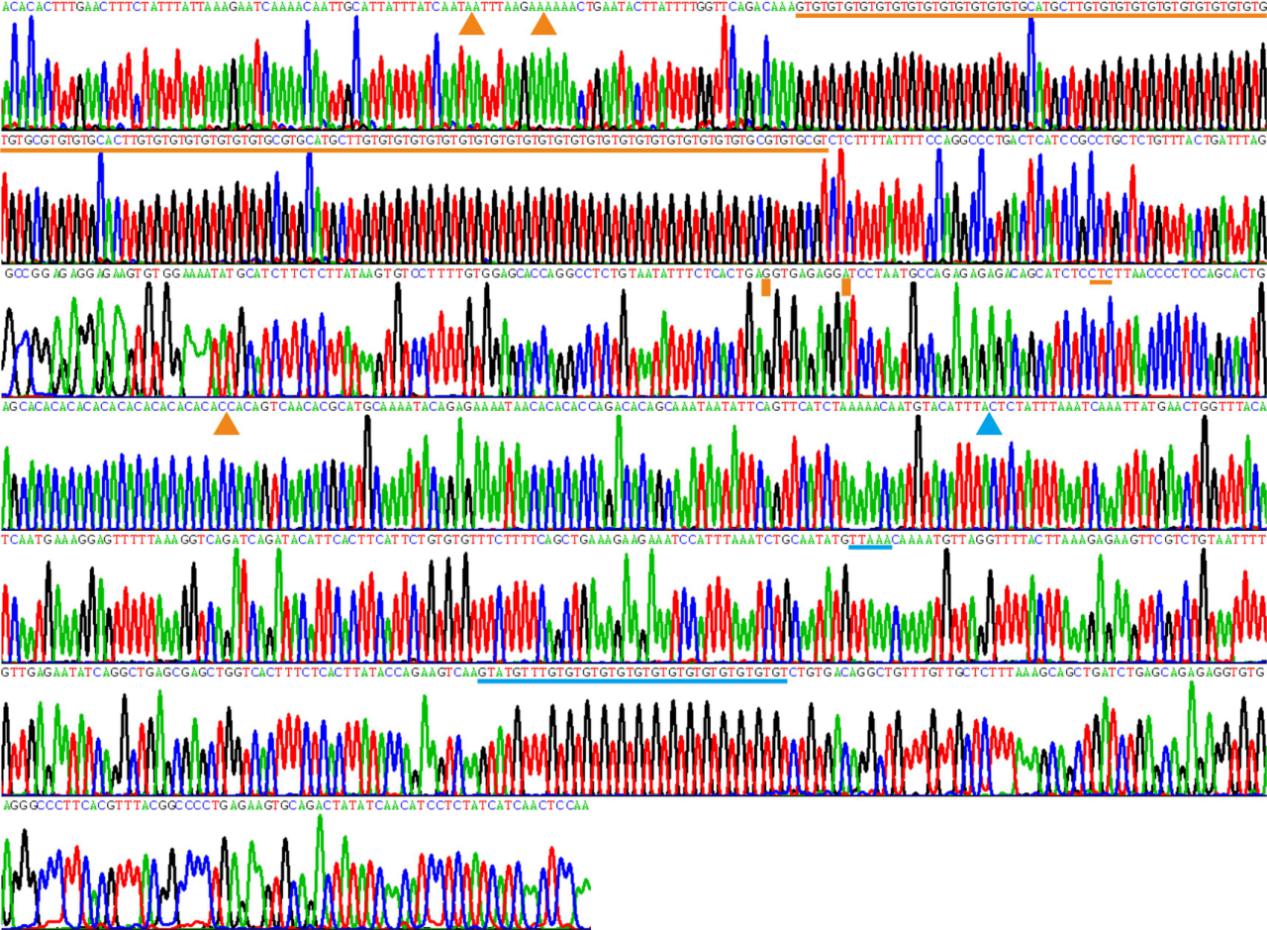


**g**


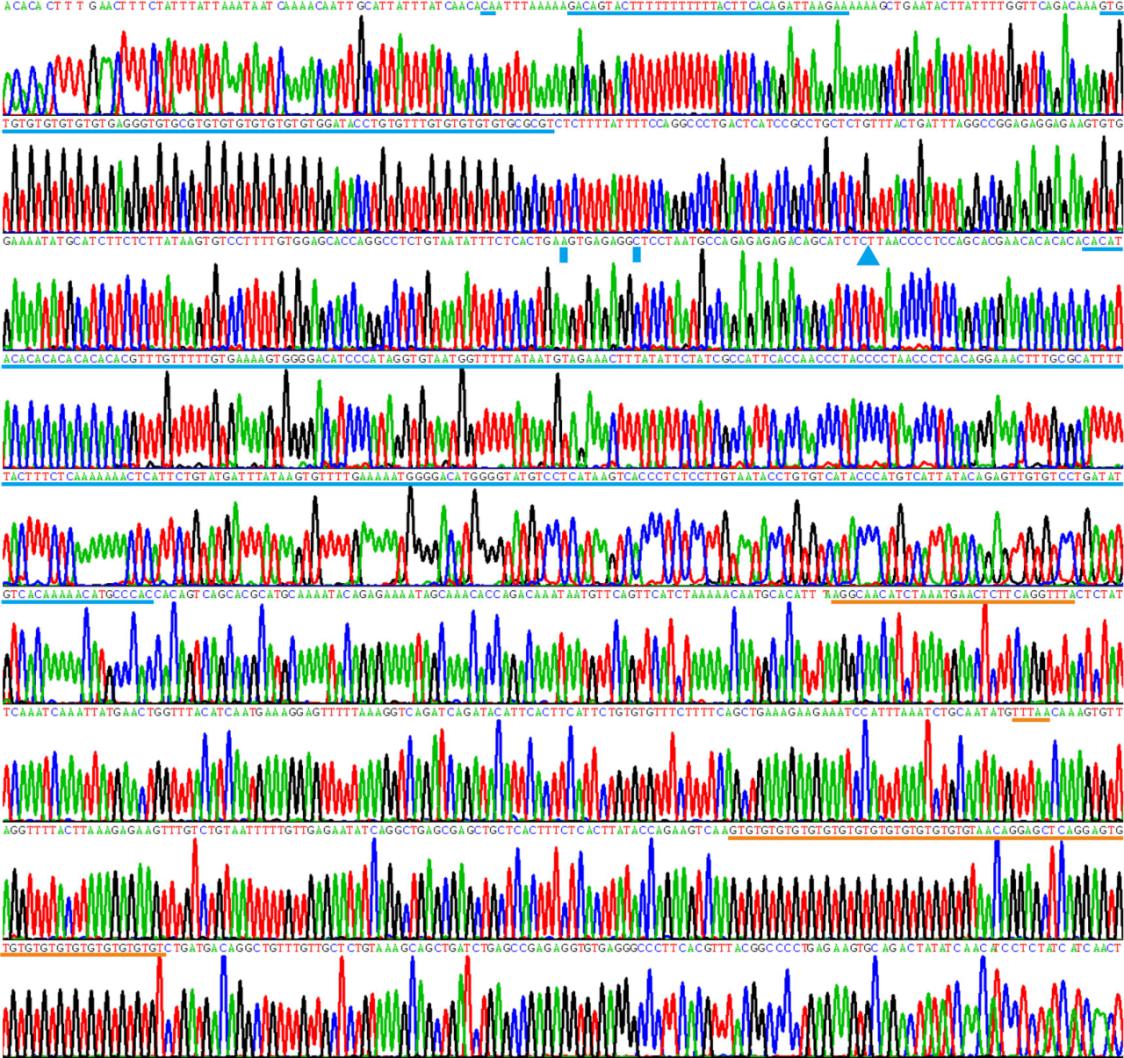


**Figure S5.** Sequencing spectrums of reciprocal crossover II clones in the region between (GT)_n_ motif 2 and 3 at different sites. Orange and blue arrow heads indicate the site a sequence being deleted in paternal and maternal genomes, respectively. Orange and blue underlines indicate the paternal and maternal specific sequences, respectively. Orange and blue boxes indicate the paternal and maternal specific SNP sites, respectively. **a**, **b**, **c**, **d**, **e**, **f** and **g** corresponds to the range of crossover II clones, respectively, in Fig. 4c.

**a**


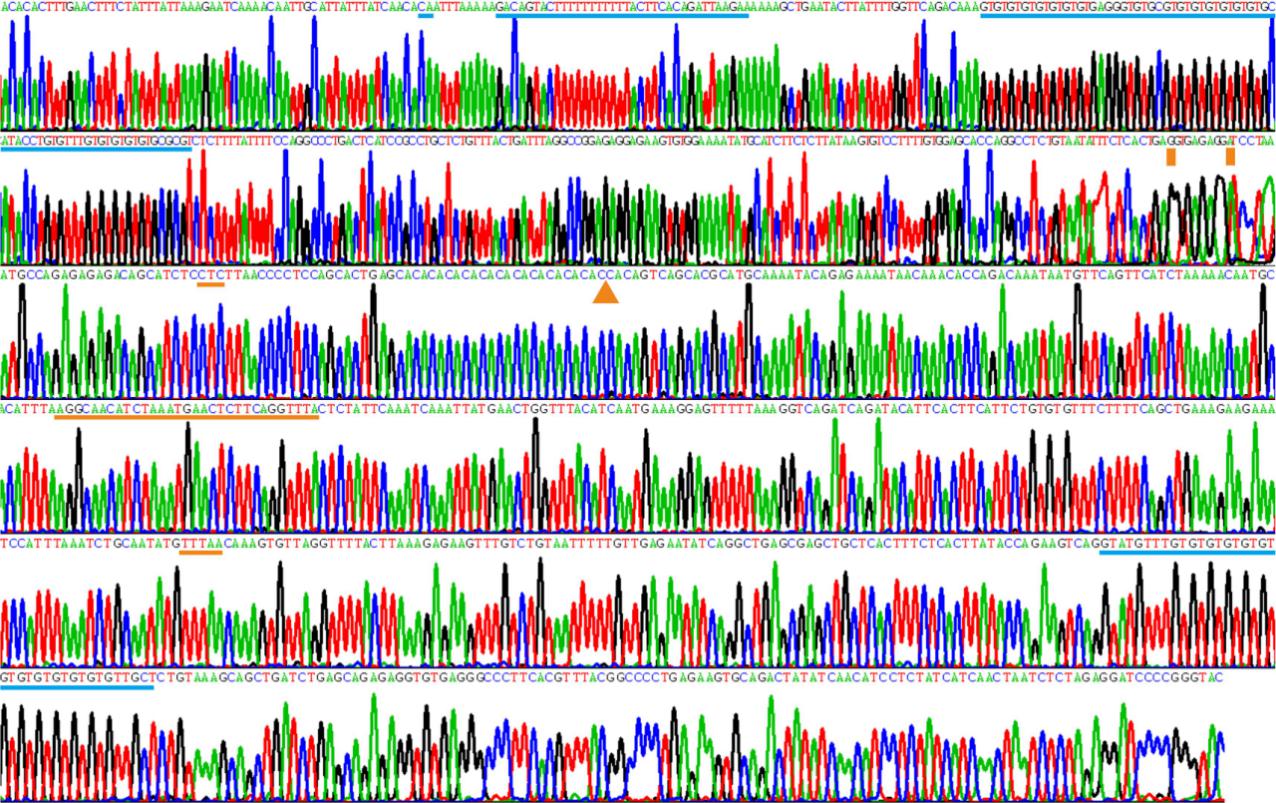


**b**


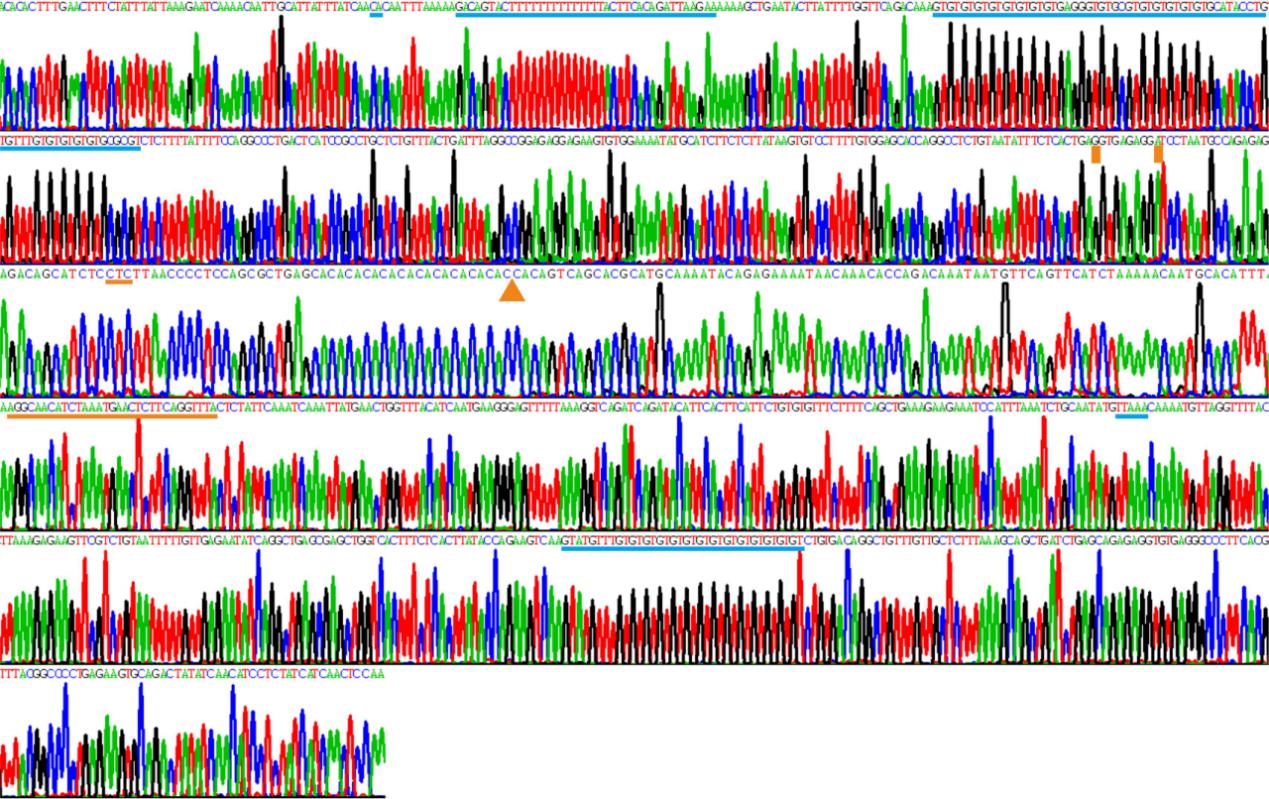


**c**


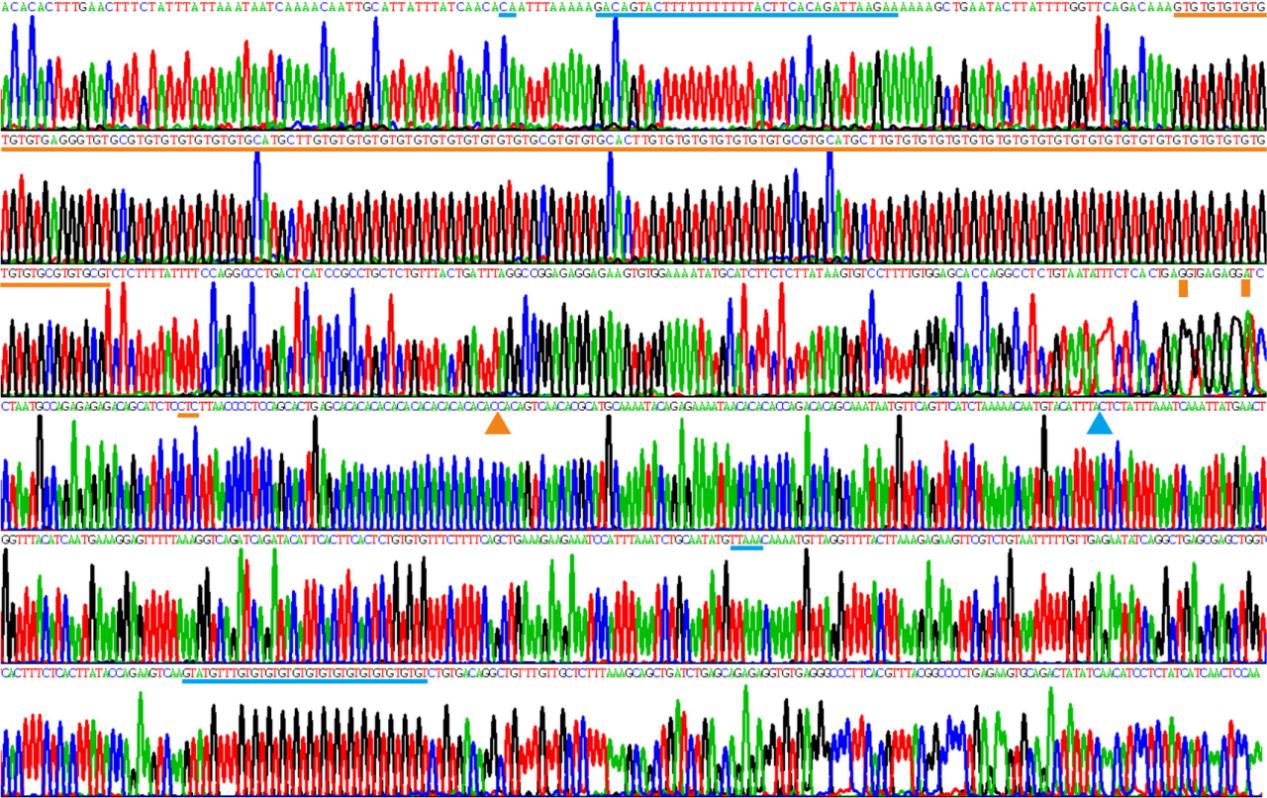


**d**


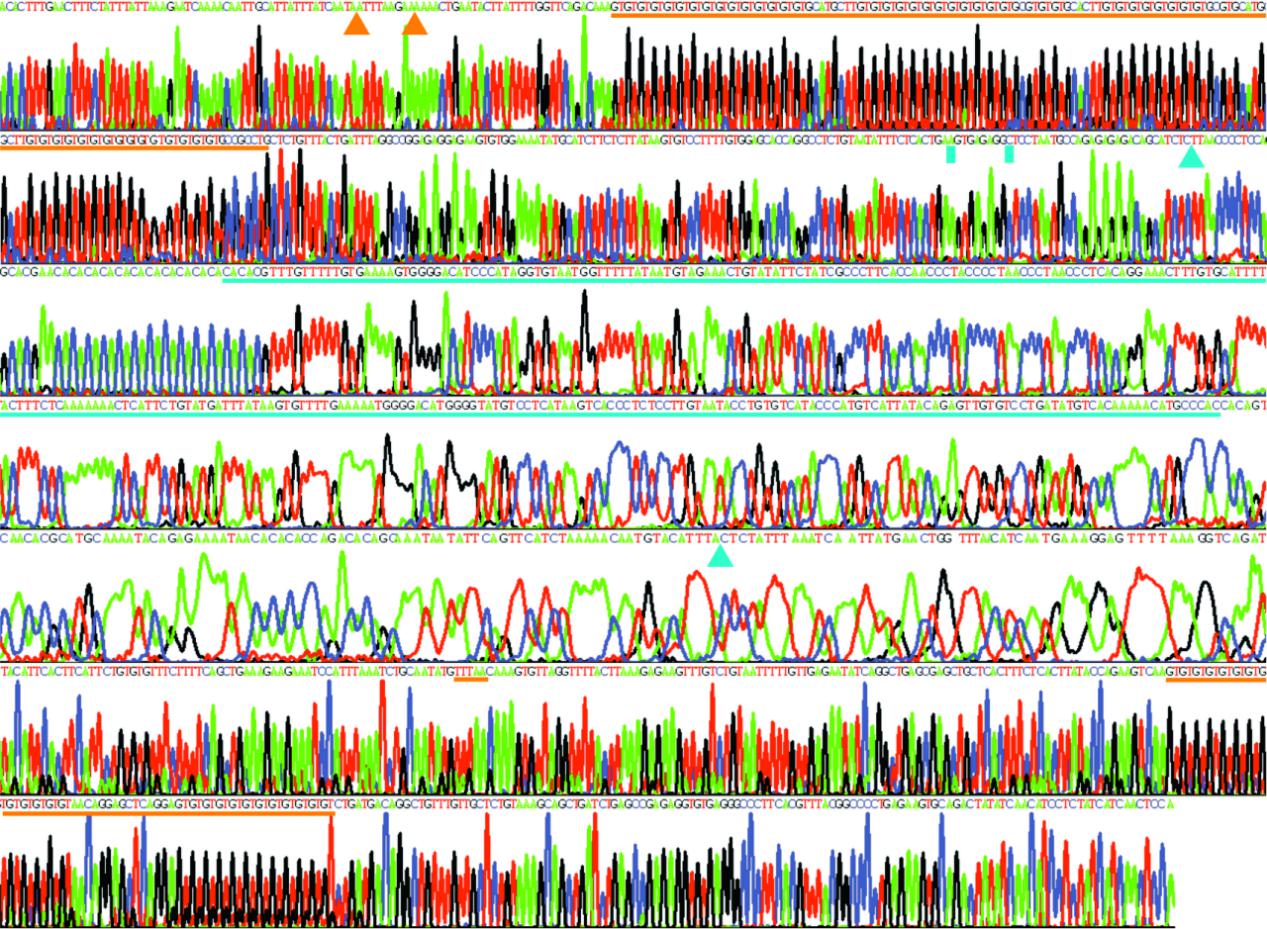


**Figure S6** Sequencing spectrums of HR clones that only a fragment is exchanged between the paternal and maternal homologous chromosomes at different sites of the (GT)_n_ motif region. Orange and blue arrow heads indicate the site a sequence being deleted in paternal and maternal genomes, respectively. Orange and blue underlines indicate the paternal and maternal specific sequences, respectively. Orange and blue boxes indicate the paternal and maternal specific SNP sites, respectively. **a**, **b**, **c** and **d** corresponds to the range of crossover I + II HR clones, respectively, in Fig. 4c.
